# The structural basis for regulation of the glutathione transporter Ycf1 by regulatory domain phosphorylation

**DOI:** 10.1038/s41467-022-28811-w

**Published:** 2022-03-11

**Authors:** Nitesh Kumar Khandelwal, Cinthia R. Millan, Samantha I. Zangari, Samantha Avila, Dewight Williams, Tarjani M. Thaker, Thomas M. Tomasiak

**Affiliations:** 1 Department of Chemistry and Biochemistry, University of Arizona, Tucson, AZ 85721 USA; 2 Department of Biochemistry and Biophysics, University of California – San Francisco, San Francisco, CA 94158 USA; 3 Eyring Materials Center, Arizona State University, Tempe, AZ 85287 USA; 4 Present Address: Duke University School of Medicine, Durham, NC 27710 USA

**Keywords:** Cryoelectron microscopy, Permeation and transport

## Abstract

Yeast Cadmium Factor 1 (Ycf1) sequesters heavy metals and glutathione into the vacuole to counter cell stress. Ycf1 belongs to the ATP binding cassette C-subfamily (ABCC) of transporters, many of which are regulated by phosphorylation on intrinsically-disordered domains. The regulatory mechanism of phosphorylation is still poorly understood. Here, we report two cryo-EM structures of Ycf1 at 3.4 Å and 4.0 Å resolution in inward-facing open conformations that capture previously unobserved ordered states of the intrinsically disordered regulatory domain (R-domain). R-domain phosphorylation is clearly evident and induces a topology promoting electrostatic and hydrophobic interactions with Nucleotide Binding Domain 1 (NBD1) and the Lasso motif. These interactions stay constant between the structures and are related by rigid body movements of the NBD1/R-domain complex. Biochemical data further show R-domain phosphorylation reorganizes the Ycf1 architecture and is required for maximal ATPase activity. Together, we provide insights into how R-domains control ABCC transporter activity.

## Abstract (web summary)

Ycf1, a C-family member ATP Binding Cassette (ABC) transporter, transports glutathione and glutathione-metal complexes in yeast. Here the authors use cryo-EM and biochemical analysis to show how an intrinsically-disordered regulatory domain (R-domain) controls activity upon phosphorylation by engaging with a Nucleotide Binding Domain.

## Introduction

ATP-binding cassette (ABC) transporters regulate the movement of diverse molecules to support fundamental roles of the membrane including lipid homeostasis, ion transport, and detoxification. The Yeast Cadmium Factor 1 (Ycf1) performs such a protective function by transporting the tripeptide glutathione, the main non-protein thiol in cells, upon oxidation to maintain redox balance or after conjugation to toxic heavy metals such as cadmium, mercury, or lead into vacuoles^1–5^. These metals make up three of the top ten environmental toxins as defined by the CDC (#s 1, 3, and 7 - https://www.atsdr.cdc.gov/spl/index.html#2019spl), marking Ycf1 as an attractive bioremediation target. Ycf1 also acts as a major redox sink and regulates redox levels in cytoplasm by sequestering glutathione after it is oxidized. In this way, Ycf1 serves a powerful protective function against metals and ROS, both of which are electrophiles that attack DNA, proteins, and the cell membrane.

**Fig. 1:**
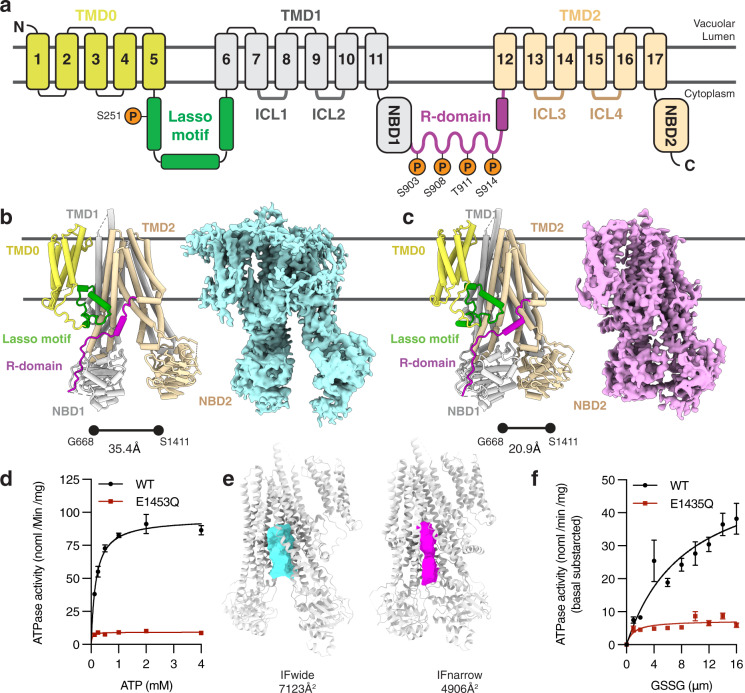
The regulatory architecture of Ycf1 determined by cryo-EM. **a** Schematic of Ycf1 structural topology highlighting the arrangement of transmembrane helices belonging to transmembrane domain 0 (TMD0, yellow), transmembrane domain 1 (TMD1, gray), and transmembrane domain 2 (TMD2, wheat). Conserved cytoplasmic elements shown include the Lasso motif (green), the R-domain (purple), nucleotide binding domains 1 and 2 (NBD1 (gray); NBD2 (wheat)) and intracellular loops 1–4 (ICL1, ICL2 (dark gray); ICL3, ICL4 (brown)). Orange spheres corresponding to conserved putative phosphorylation sites in Ycf1 are labeled with their residue numbering on the corresponding domains. Cryo-EM model (left) and map (right) of the **b** IFwide (cyan) and **c** IFnarrow (pink) conformations of E1435Q Ycf1 colored using the same scheme as shown in (**a**). **d** ATPase activity in the presence of increasing concentrations of ATP for wild-type (WT) and the catalytically dead (E1435Q) variant of Ycf1. **e** Volume representation of the internal cavities of IFwide (left, cyan) and IFnarrow (right, pink). **f** ATPase activity in WT and E1435Q Ycf1 in the presence of increasing concentrations of oxidized glutathione (GSSG) and 1 mM ATP. Data are reported as stimulated rates in which the basal ATPase activity (without GSSG) was subtracted. Data shown correspond to a half-maximal effective concentration (EC_50_) of 8.7 ± 2.6 µM for GSSG-induced stimulation of ATPase activity in WT Ycf1. Results in (**d**) and (**f**) are the mean ± the standard deviation (SD) for *n* = 3 (technical triplicates). Source data are provided as a Source Data file.

Because of their essential roles in responding to cell stress, such proteins are often tightly regulated. Protein phosphorylation represents one such mechanism by which protein and transporter function can be tuned for specific cellular contexts. In general, approximately half of all human ABC transporters are phosphorylated^6^ including the medically important P-glycoprotein^7^, the Sulfonyl Urea Receptor (SUR1^8^), and the Cystic Fibrosis Transmembrane conductance regulator (CFTR^9^). Rapid regulation is especially important to the C-subfamily of ABC transporters (ABCC family), which includes Ycf1 and CFTR, in particular because of their roles in regulating osmotic balance, neutralizing reactive oxygen species, or combating the effects of antifungal or anticancer agents in many organisms. Often, transporter phosphoregulation is mediated through phosphorylation on regions outside of the highly structurally conserved transport machinery. Instead, phosphorylation sites arise in long, disordered loops between domains or at the termini. In ABCC transporters, such regulatory elements are evolutionary additions to the canonical ABC exporter fold composed of two transmembrane domains (TMDs) and two nucleotide-binding domains (NBDs) (Fig. 1a). These additions include an accessory TMD called TMD0, a Lasso motif that connects TMD0 and TMD1, and a ~60 to 140 amino acid disordered domain termed the regulatory domain (R-domain) that connects NBD1 and TMD2^10–13^. The R-domain in particular acts as a signaling and interaction hub in ABCC transporters and contains multiple PKA, PKC, and CKII recognitions sites with otherwise little sequence conservation.

Disruption of this regulatory function can have profound cellular effects. Mutations of R-domain phosphorylation sites lead to loss of cadmium tolerance in Ycf1^11^ and to lower open probability (but not overall open duration) and sensitivity to ATP concentration in the Ycf1 homolog CFTR^14,15^. Furthermore, mutations in 19 of the total ~130 residues of the CFTR R-domain are associated with mutants in the CFTR-linked lung disease, cystic fibrosis (www.cftr2.org). Phosphorylation can induce negative regulation as well, particularly on the Lasso motif in both Ycf1^16^ and the Ycf1 functional homolog Multidrug Resistance Protein 1 (MRP1^17^). Finally, the R-domain provides a surface for protein-protein interactions, including to other transporters such as SLC26A3 and the adapter protein 14-3-3β^18^.

Despite recent advances in ABC transporter structural biology by cryo-electron microscopy (cryo-EM), the biophysical basis of these diverse R-domain interactions is poorly understood. Barriers to these characterizations hinge on the nature of the R-domain, which is predicted to be unstructured based on low sequence conservation^19^ and experimentally confirmed with circular dichroism^19,20^ and proteolysis analysis that estimate as low as 5% helical content^20^. However, more recent NMR experiments point to discrete helical fragments in the R-domain and direct interactions with NBD1^21^. Meanwhile, cryo-EM structures of ABCC1, CFTR, and Ycf1 reveal a mostly disordered R-domain^22–24^ without assigned sequence for the phosphorylation sites, limiting our understanding of R-domain interactions. Structures of human CFTR and Ycf1 have revealed density for segments of the R-domain in proximity to NBD1^25^. Unfortunately, the lack of a continuous R-domain sequence assignment in these models, especially in the phosphorylated regions, prohibits insights into the interfaces that govern R-domain regulation^25^.

In this study, we have used cryo-EM to determine the 3.4 and 4.0 Å resolution structures of endogenously phosphorylated *Saccharomyces cerevisiae* Ycf1 in two inward-facing conformations: wide (IFwide) and narrow (IFnarrow). We observe a resolved section of the R-domain that is most highly conserved in the ABCC family and includes the phosphorylation sites (Fig. 1b, c and Supplementary Table 1). Our structures reveal the extensive interaction interface between the phosphorylated R-domain, NBD1, and the Lasso motif. Interestingly, the networks we observe are nearly constant between the two states, related only by a rigid movement of the entire R-domain/NBD1 complex relative to NBD2, with very minor changes in the positioning of the R-domain along NBD1. This positioning is almost entirely replicated in contacts predicted by an AlphaFold2 analysis. Biochemical investigation confirms this positioning and shows that phosphorylation induces changes in ATPase activity at the same time as it causes structural changes. Thus, we provide the structural and biochemical details of phosphorylation-dependent regulation of Ycf1.

## Results

### Cryo-EM structures of inward-facing wide and narrow states of Ycf1

In asymmetric ABC transporters, like Ycf1, only one of two NBDs (NBD2) is capable of efficient ATP hydrolysis. To enable structural studies of Ycf1, we expressed and purified two forms in the *S. cerevisiae* DSY5 strain: wild-type (WT) and the NBD2 Walker B mutant E1435Q (Supplementary Fig. 1). Grids of both WT and E1435Q Ycf1 in apo conditions were prepared as described in the methods, but only the E1435Q Ycf1 dataset yielded homogenous particles from which we successfully identified not one but two inward-facing conformations determined to 3.4 and 4.0 Å resolution (Fig. 1b, c). The E1435Q mutant resulted in substantially lowered ATPase activity compared to WT Ycf1 (V_max_ 95 ± 2 nmol/min/mg) (Fig. 1d), likely allowing the trapping of these conformations. Both maps were sufficiently detailed to enable modeling of an almost complete Ycf1 protein structure. The features we observe are largely consistent with other ABCC structures^22,26,27^. This includes 17 transmembrane helices of TMD1 and TMD2, the accessory transmembrane domain TMD0 unique to the ABCC family, the Lasso motif, both NBDs, and surprisingly a major portion of a previously uncharacterized region of the R-domain (Fig. 1a–c).

The two states are defined by clear differences in the interdomain distances between the NBDs (Fig. 1b-c) and the TMDs that define the substrate-binding cavity (Fig. 1e). We designated the state in which the NBDs are further apart as IFwide (Fig. 1b) and the state in which the NBDs are closer as IFnarrow (Fig. 1c). The unoccluded substrate cavities in both structures are large enough to accommodate oxidized glutathione (GSSG) (Fig. 1e), previously shown to be a primary physiological substrate in cells^5^. These results are consistent with our ATPase data that show GSSG indeed stimulates ATPase activity at physiologically relevant concentrations (µM concentration range) (Fig. 1f and Supplementary Fig. 1e).

### The Ycf1 R-domain is in a phosphorylated state with an ordered regulatory domain

**Fig. 2:**
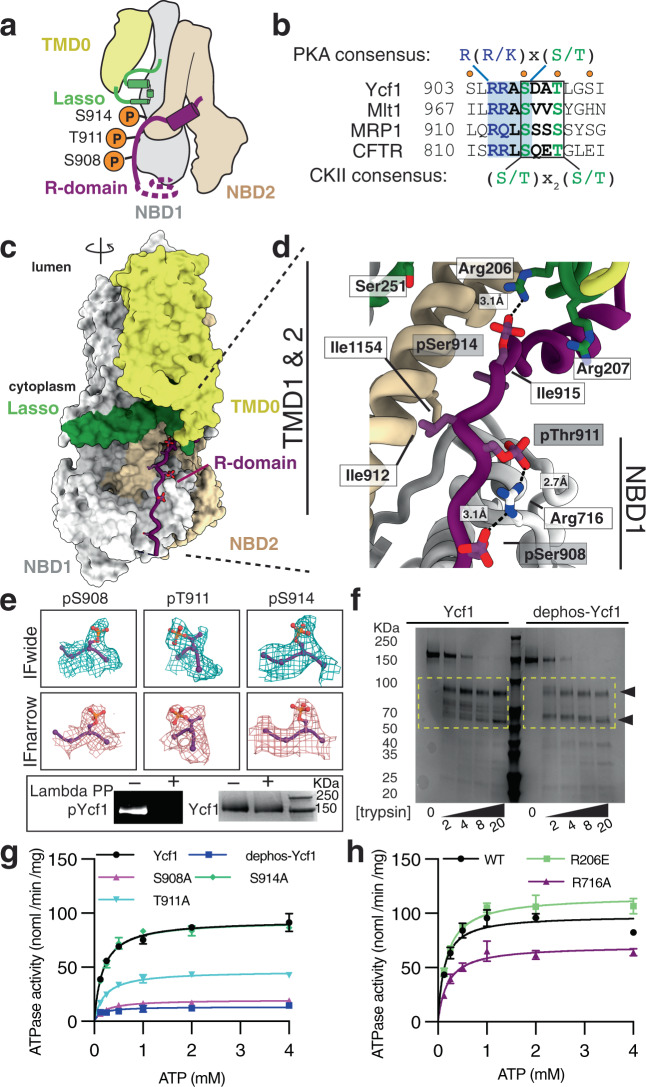
The R-domain engages Ycf1 through an extensive phosphorylation-dependent network. **a** Putative phosphorylation sites on the Ycf1 R-domain (purple) mapped onto a cartoon representation of the Ycf1 IFnarrow cryo-EM structure. **b** Sequence alignment of ABCC family members highlighting the consensuses sequences of putative phosphorylation motifs. Orange dots represent predicted phosphorylation sites. **c** Surface representation of the cryo-EM model of IFnarrow Ycf1 highlighting the orientation of the R-domain along NBD1. **d** A detailed view of the R-domain region shown in (**c**) highlighting residues contributing to the binding interface between the R-domain (purple), the Lasso motif (green), and NBD1 (gray). Carbon atoms are colored consistent with domain coloring in (**c**), with oxygen (red), nitrogen (blue), and phosphate (orange) atoms colored accordingly. Dashes represent hydrogen bonds or electrostatic interactions between heavy atoms. **e** Electron potential density of phosphorylated residues (S908, T911, and S914) observed in IFwide (cyan) and IFnarrow (pink) upper panel. SDS-PAGE analysis of phosphorylation in purified samples of Ycf1 in the presence or absence of Lambda phosphatase (Lambda PP) treatment. The left gel showing phosphorylated Ycf1 (pYcf1) was stained with Pro-Q phosphoprotein gel stain (Thermo Fisher), whereas the right gel was visualized with Coomassie gel stain to show total Ycf1 (Ycf1), Experiment is performed in biological replicate and representative gel is shown. **f** Representative SDS-PAGE analysis of proteolysis resistance in Lambda PP untreated (Ycf1) or treated (dephos-Ycf1) Ycf1 incubated with increasing concentrations of trypsin (0–20 µg/mL). The experiment was performed in biological replicate **g** ATPase activity of phosphorylated (Ycf1), dephosphorylated (dephos-Ycf1) and phosphorylated residue mutants (S908A, T911A, and S914A). Data shown are the mean ± SD for *n* = 4 (technical quadruplicates). Source data are provided as a Source Data file. **h** ATPase activity of the R716 mutant variant R716A and the R206 mutant variant R206E along with WT Ycf1. Data shown are the mean ± SD for *n* = 3 (technical triplicates). Source data are provided as a Source Data file.

**Fig. 3:**
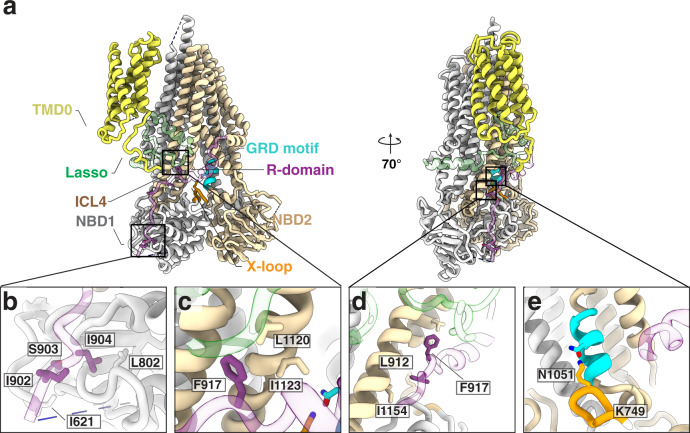
R-domain interaction network in Ycf1. **a** Overall structure of Ycf1 in IFnarrow. **b**–**d**. Hydrophobic pockets along the R-domain/NBD interface. **e** Interactions between residues of the X-loop and GRD motif in IFnarrow. The R-domain is colored purple, TMD1-NBD1 is colored gray, TMD2 and NBD2 are colored wheat, Lasso motif is colored green, the X-loop is colored orange, and the GRD motif is colored cyan in all figures.

The most striking findings of our structures are the visualization of every regulatory element, including the R-domain (residues 901-935) phosphorylated in three positions. The R-domain is helical (residues 920–929) in the C-terminal segment and lacks secondary structure (residues 901–919 and 930–935) in the other. The C-terminal helix was key to sequence assignment and aligns with partial models from CFTR cryo-EM structures (Supplementary Fig. 7a–e,^25^), with helical NMR assignments in CFTR^21^, and with evolutionary coupling analysis of co-evolving residues^28^ from a multisequence alignment of 13,723 homologs (Supplementary Fig. 7f). The R-domain encircles NBD1 in both IFwide and IFnarrow (Fig. 1b, c) and is stabilized by extensive contacts along a basic groove of the NBD1 perimeter, intracellular loop 4, and the Lasso motif (Figs. 2a–d and 3). This positioning differs from the positioning and sequence assignment of the R-domain fragments in a previous Ycf1 structure^27^. Finally, a second regulatory region, the regulatory insertion (residues 615-643), interacts with the R-domain through an electrostatic or H-bond interaction from K617 to S903, another residue that is known to be phosphorylated (Supplementary Fig. 7i).

The R-domain houses several phosphorylation motifs shown to be important for regulation (Fig. 2b). We assigned phosphorylation to S908, T911, and S914 in our model based on pronounced additional electron potential density at these residues (Fig. 2e upper panels). Phosphostaining analysis (Fig. 2e lower panels) and mass spectrometry (Supplementary Table 2 and Supplementary Fig. 8) confirm phosphorylation at these three sites in Ycf1. These phosphates make extensive contacts with the Nε and Nη atoms in the guanidyl groups of R716 on NBD1 and R206 on the Lasso motif in proximity to be either hydrogen-bonding or strong charge–charge interactions (Fig. 2d). Among the phosphosites, S908 is part of a recognition motif for PKA-like kinases called the RRAS motif (or dibasic consensus sequence) and displays a similar conformation to that of a phosphomimetic peptide bound to PKA (PDB ID: 1ATP^29^). T911 also serves as part of a CKII kinase recognition motif (S/T-(x)_n_-S/T) that partially overlaps with the PKA-like kinase site (Fig. 2b). Overall, the resolvable range of the Ycf1 R-domain coincides with the region containing the highest sequence conservation to CFTR, which possesses a longer R-domain than Ycf1 but otherwise conserved kinase motifs (Fig. 2b and Supplementary Fig. 7e).

### Phosphorylation of the R-domain controls dynamics and ATPase activity

The R-domain interaction with NBD1, TMDs, and Lasso motif has been hypothesized to be highly dynamic and dependent on phosphorylation of the R-domain. To interrogate this effect on our reconstituted sample, we prepared dephosphorylated Ycf1 by treating it with lambda phosphatase (Fig. 2e) and subjecting the sample to limited proteolysis with trypsin. We observed that dephosphorylated Ycf1 substantially changes the digestion pattern in comparison to endogenously phosphorylated Ycf1 (Fig. 2f), with a clear increase to protease accessibility in the dephosphorylated state as judged by a more complete digestion (ie loss of intermediate-sized bands) in the dephosphorylated state. We reasoned these differences result from changes in the R-domain position or conformation in a phosphorylation-dependent manner similar to CFTR^25^ but note that the phosphates themselves may block protease accessibility to some sites as well.

To investigate the functional impact of these structural changes due to dephosphorylation, we interrogated changes in ATPase activity. ATPase assays show a drastic activity reduction in dephosphorylated Ycf1 (Fig. 2g) comparable to mutation of the ATPase site, suggesting that the interactions of phosphorylated S908, T911, and S914 are major drivers of an architecture that facilitates ATP hydrolysis.

### Mutations of the R-domain-NBD1 interface interfere with ATPase activity

Ycf1 contains three R-domain phosphorylation sites (S908, T911, and S914) that were captured in our structures. To pinpoint which sites are most important for activity, we generated mutants of the phosphorylated residues (S908, T911, and S914) and important interacting residues (R206 and R716) and performed ATPase assays. The S908A mutant causes drastically lowered (~80% loss) ATPase activity and the T911A mutant causes ~50% loss in activity. Surprisingly, no change in ATPase activity in the S914A mutant was detected (Fig. 2g). This pattern is consistent with overall conservation. S908 and T911 are highly conserved sites among other homologs and are part of the PKA and CKII kinase consensus sites (Fig. 2b) while S914 is not conserved. Cell viability data also show the same pattern where *S. cerevisiae* cells with Ycf1 S908 and T911 mutations show deficient cadmium detoxification, resulting in cell death^11^ while S914 mutation did not impact the function of Ycf1^10^.

Next, we investigated the interaction of these phosphorylated residue with their interacting partner residues (R716 and R206) (Fig. 2d). S908 and T911 form a prominent hydrogen bonding or salt bridge interaction with R716. The R716A mutant shows loss of ATPase activity (~30% loss) (Fig. 2h) consistent with S908A and T911A mutants but to a lesser degree. It is unclear why the R-domain mutants showed a higher loss of activity, but it is possible there are other compensating interactions between the phosphorylated R-domain and the overall positive charge of NBD1. This supports our observation that the presence of positive/polar charged residues at this position interacting with negatively charged phosphorylation sites in the R-domain is important for proper functioning of this family of transporters (Supplementary Fig. 9a).

Conversely, although S914A mutation did not impact ATPase activity, we investigated the impact of its interaction with R206 (Fig. 2d). We generated the R206A mutant variant, but this mutation leads to highly unstable protein. We instead made the R206E mutant and tested for ATPase activity. Similar to its interacting partner (S914), the R206E mutant did not show any negative impact on ATPase activity but surprisingly we observed slightly increased ATPase activity (Fig. 2h). Consistent with this observation and with its interacting partner S914, we did not observe sequence conservation at this site (Supplementary Fig. 9b).

The overall pattern of deleterious (S908A/T911A + R716A) and neutral or marginally positive (S914A + R206E) mutations aligns with the structural placement of the phosphorylated R-domain. We generated a double mutation of the R-domain (S908A/T911A) to investigate full attenuation of phosphorylation sites, but this resulted in highly destabilized protein that could not be purified. These results suggest that the observed interactions in our structure exhibit functional consequences when mutated and that partial compensation of single R-domain mutants by other phosphorylation sites can maintain some activity. The lack of stability of the double mutant suggests the complete loss of the R-domain-NBD1 interface.

### Tightly embedded hydrophobic surfaces stabilize the R-domain NBD interaction

Together, the phosphorylation sites form a scaffold that engages NBD1 and the Lasso motif along a single axis to connect NBD1 and the TMDs (Fig. 3a). The electrostatic interactions are supported by a substantial burying of hydrophobic surfaces at 3 sites (site 1: I621 - L802 - I902 - I904 (Fig. 3b); site 2: F917 - I1123 - L1120 (Fig. 3c); and site 3: L912 - I915 - I1154 (Fig. 3d)). These interactions are poised to provide a large thermodynamic driving force of R-domain binding and stabilize the R-domain/NBD1 interface, burying ~1550 Å^2^ and ~1283 Å^2^ of surface area along the entire R-domain in IFnarrow and IFwide, respectively (~546 Å^2^ (IFnarrow) and ~425 Å^2^ (IFwide) specifically between NBD1 and the R-domain) (Fig. 2c). In addition, this region overlaps with known allosteric motifs, including the X-loop and the GRD motif^30^, both of which also form contacts in IFnarrow (Fig. 3e).

### Positioning of the R-domain is almost entirely captured by AlphaFold2 analysis

To verify the Ycf1 R-domain position, we used the new AlphaFold2 structure prediction algorithm that has recently scored a substantial improvement in the Critical Assessment of the Structures of Proteins (CASP) contest^31^. Analysis of the Ycf1 prediction shows a close recreation of the cryo-EM structures shown here, with a strikingly similar organization of the R-domain as the IFwide and IFnarrow structures. The R-domain region from 904-914 matches the ϕ−ψ backbone distribution and side-chain positions almost exactly, with several identical rotamer positions (Supplementary Fig. 10). This region covers the three phosphorylation sites in Ycf1 and recreates the same charged and hydrophobic interactions described above. Critically, this region contains the highest predicted Local Distance Difference Test (pLDDT) scores (the degree of confidence of an amino acid and all surrounding amino acids) throughout the R-domain. The highest pLDDT value of 70 is found in D909 (on a scale of 0–100), which corresponds to a ~0.7 probability of correct ϕ−ψ angles.

The AlphaFold2 analysis also recreates the intersection of the Lasso motif, R-domain, and the loop following TMD0 observed in our structures. Lower confidence regions show some divergence between the two structures, namely from 916–930, although both represent part of this region as α-helical. This region encompasses a clearly visible phenylalanine (F917) in the cryo-EM structure, but a movement of the downstream region of the α-helix, possibly due to a different overall conformation of the entire structure. Furthermore, the AlphaFold2 structure recreates clearly visible cryo-EM density for almost the entirety of the R-domain residues 883–897 and 849–860, excluding ~20 residues; however, the position of these residues cannot be built with confidence and was excluded from the final model (Supplementary Fig. 11).

### Location of an inhibitory phosphosite at S251 in proximity to R-domain phosphosites

The Lasso motif region adjacent to the R-domain also contains a conserved phosphorylation site that negatively regulates transport, S251 (T249 in the Ycf1 homolog MRP1)^16,17^. Though we do not observe phosphorylation in our structure at this position, S251 is accessible to the surface and poised to disrupt R-domain interactions through electrostatic repulsion of phosphate groups in the R-domain (Fig. 2d). Thus, our structures provide a plausible explanation of the negative impact of the Lasso motif phosphorylation on Ycf1 function.

### The two structures of Ycf1 show different NBD orientations

**Fig. 4:**
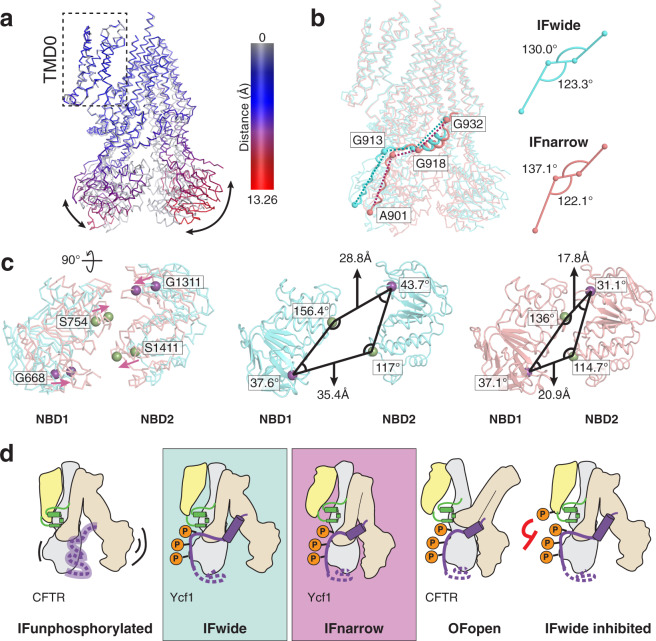
Proposed model for catalytic control in Ycf1 relies on transitions of the rigid R-domain architecture from IFwide to IFnarrow. **a** Overlay of IFnarrow (gray) and IFwide states of Ycf1, in which IFwide is colored by RMSD calculated on a per residue basis from structural alignment between TMD0 of each state. **b** Comparison of the R-domain geometry and differences observed for the intradomain angles between flexible residues forming hinge regions in the R-domains in each state. **c** Superposed NBDs (left panel) from IFwide (cyan ribbon) and IFnarrow (pink ribbon) highlighting the rearrangement in the relative positions of conserved residues of the signature motif (S754 and S1411, green sphere) and the walker A motif (G668 and G1311, purple spheres) in each state. The specific interdomain angles formed by these sites in their respective states are shown in the middle (IFwide) and right (IFnarrow) panels. **d** Proposed model for organization and regulation of the R-domain through transport in light of the IFwide and IFnarrow Ycf1 structures and in the context of previously published CFTR structures (IFunphosphorylated (PDB ID: 5UAK^26^); OFopen (PDB ID: 6MSM^25^). Domains are colored yellow for TMD0, gray for TMD1 and NBD1, wheat for TMD2 and NBD2, green for the Lasso motif, and purple for the R-domain.

The observed R-domain–NBD1–lasso contacts are consistent between IFwide and IFnarrow with minimal changes between these domains (Fig. 4a). Instead, a rearrangement within the R-domain correlates with overall movements in the structure, including a change in angle between residues G913, G918, and G932 of the R-domain from 130° (IFwide) to ~137° (IFnarrow) (Fig. 4b). Both G918 and G932 cap the helical R-domain region and are poised to act as a hinge for the movement of this structured region between the transition from IFwide to IFnarrow.

In contrast to the interaction sites themselves, the major structural consequences of the IFwide to IFnarrow transition are induced at the ATP-binding sites. These sites not only move closer in IFnarrow but also rotate to occlude the non-functional ATP-binding site in NBD1 (Fig. 4c). Notably, the second site in NBD2 (active) is open for ATP binding. NBD2 is the less ordered of the two NBDs as inferred from relatively weaker cryo-EM density in this region in both states. This disorder is a predominant feature also noted for a third, less well-resolved state that we call IFtransition (Supplementary Fig. 3c) which was insufficiently detailed to enable model building. Concurrently, several coupling elements that connect the NBDs and TMDs, including the NBD1 X-loop and GRD motif^30^, are brought into contact upon transition to IFnarrow (Fig. 3e). Overall, these architectural changes resemble changes in substrate-bound and substrate-free states of the Ycf1 homolog MRP1 (Supplementary Fig. 12a-f)^22,32^. These movements support a model where R-domain phosphorylation helps stabilize NBD1 to recruit other domains into a conformation (IFnarrow) suitable for ATP binding and initiation of transport. We propose this yields a primed, active state ready for transport.

## Discussion

The structures of Ycf1 determined here reveal previously unobserved interactions in the ABCC family of ABC transporters that inform on how they are regulated. This includes an inward-open conformation with an intact and endogenously phosphorylated R-domain tightly bound to NBD1. Our structures provide a more complete model than previous ABCC structures, which resolve small fragments of the R-domain, do not resolve the phosphorylation sites, and/or do not assign the sequence for this region^23,25,27^. The presence of an ordered R-domain position poses a surprise, since many alternate models exist for the R-domain mechanism that include a completely disordered R-domain or an R-domain that resides only between NBD1 and NBD2 in a semi-ordered state.

The findings in this work support and clarify decades of biochemical, cellular, and clinical data on the consequences of R-domain interactions. The multiple phosphate-binding interactions to a common surface along NBD1 and the Lasso motif explain the well-known robustness to loss of any single phosphorylation site on the R-domain^9,11^ and the requirement for an intact N-terminus containing the Lasso motif of CFTR for activity^33^. The extensive R-domain and NBD1 interactions increase the ATPase rate in a manner consistent with CFTR, where R-domain phosphorylation potentiates ATP binding and hydrolysis but otherwise does not induce transport activity alone^34^. Our structures also are consistent with the visible R-domain density in CFTR and polyalanine models built into this density (Supplementary Fig. 7a–d).

The position of the R-domain informs on the phosphorylation-dependent activation mechanism and provides clarification on the competing models that exist. In the most widely held model, CFTR activation depends on release of the R-domain positioned between NBD1 and NBD2 upon phosphorylation to induce a completely unbound and disordered state that releases the NBDs to allow dimerization^26^. Our findings here do not support this model. Instead, our results suggest that the extensive interactions between the phosphorylated R-domain and the rest of Ycf1 represent a stimulatory R-domain architecture. However, our structures do not rule out an additional inhibitory role for the unphosphorylated state (Fig. 4d). Based on our functional data, we propose a model where the R-domain transitions from unbound/disordered to bound/ordered upon phosphorylation, residing either between the NBDs when dephosphorylated^35^ or near NBD1 but loosely associated. Phosphorylation along the R-domain drives association with the periphery of NBD1 (Fig. 2c) that is reflected as both increased ATPase activity and R-domain stability (Fig. 2f–h). The tighter assembly in IFnarrow compared to IFwide may reflect the end result of the transition initiated by R-domain phosphorylation that leave key components of the transport cycle (ie NBDs, X-loop, GRD motif) engaged (Supplementary Movie 1). The clustering of the R-domain and Lasso motif also provides a plausible explanation for the inhibitory role of S251 phosphorylation, which is poised to disrupt the R-domain interaction network by charge repulsion (IFwide inhibited—Fig. 4d). In conclusion, R-domain interactions lend a regulatory effect analogous to that of a transmission “clutch” that only engages an engine but otherwise does not perform thermodynamic work itself.

The overall structures of Ycf1 that we present differ substantially from a recently published cryo-EM structure of Ycf1 (PDB ID: 7MPE^27^). The most pronounced difference is an R-domain register shift of 40 positions (901-927 in this structure compared to 860-874 in 7MPE). Outside of the R-domain, both structures match closely with a Cα RMSD of 2.3 Å and similar overall topology. It is unclear whether this is another state of the R-domain but several pieces of evidence support its placement in the model presented here. First, continuous density was visible from the end of the R-domain to the 2nd TMD that allowed for model building of our structure. This assignment allowed for placement of key residues including F917 in clear density in a hydrophobic pocket and ultimately placement of phosphorylated residues of the R-domain that closely match thos in CFTR. Second, our mutagenesis data provide consistent evidence of the functional importance of R-domain interfaces when mutated on either the R-domain or its corresponding binding surface (Fig. 2g–h).

Finally, independent support of our model comes from the AlphaFold2 algorithm. This structure shows a remarkable match of the R-domain to the cryo-EM structure down to side chain orientations and rotamer positions. The overall RMSD of full-length Ycf1 is lower between our IFwide model and the AlphaFold2 model (3.6 Å) compared to 7MPE and the AlphaFold2 model (6.3 Å). An even higher discrepancy exists between the alignments of just the NBD1 + R-domain region (610–927) performed to account for domain motions between structures (IFwide to AlphaFold2 model: 2.6 Å versus 7MPE to AlphaFold2 model: 10.7 Å). High predicted pLDDT scores (a measure of confidence in AlphaFold2) along the conserved phosphorylation motifs (>70 at D909) confirm the placement of the R-domain and suggest high sequence co-evolution in this region, particularly between positive charges on NBD1 and the phosphorylatable R-domain residues. The AlphaFold2 model also shows reasonable placement of the remaining R-domain segments, including those preceding the start of R-domain model (880–900) and part of the R-domain after NBD1 (849-859) consistent with visible electron potential density from our cryo-EM maps. Although these segments are still too disordered to reliably be modeled without another source of distance restraints, they are more consistent with the placement of the entire R-domain in our models.

It is unclear why there are such substantial differences in the placement of the R-domain. The alternate arrangement of the R-domain in the 7MPE model is physically plausible as it places a cluster of electronegative side chains where our model has placed phosphates. Because of the remaining “slack” in the R-domain, it is possible that both models are correct and represent different substates of the bound R-domain structure. Recent reports from CFTR suggest that the R-insertion can alter its secondary structure and register along NBD1^36^. This suggests that perhaps a common mechanism exists with the R-domain as well, although further biophysical and computational studies will be needed to uncover how.

Our results also rationalize key clinically relevant findings related to other ABCC family members. The extensive electrostatic, hydrogen bonding, and hydrophobic interactions on the R-domain explain how mutations distal from phosphorylation sites may nevertheless contribute to dysfunction. Additionally, the site equivalent to the most widespread cystic fibrosis linked mutant, (CFTR delF506) Ycf1 F713, is located in NBD1 directly adjacent to the phosphorylation sites of the R-domain and is coordinated by cation-pi interactions with two conserved arginine side chains, R765 and R1150 (Supplementary Fig. 7h). Interestingly, the thermostability defects in the delF506 mutants correspond to similar folding defects to the double R-domain mutants of Ycf1. Finally, the binding site of lumacaftor, a clinically used corrector that stabilizes unfolded CFTR, binds in the outer perimeter of NBD1 near the R-domain binding site^37^. The high convergence of several important effects near one region where the R-domain, NBD1, R-insertion, and Lasso motif come together suggest the general importance of the R-domain interactions observed in our structures.

In summary, our structures provide mechanistic insights into post-translational modification (phosphorylation) of an important class of transporters, the ABCC family. The presence of a single defined binding site for the conserved part of the R-domain confirms an anticipated but elusive binding site and may provide implications for several other types of transporters where such long phosphorylated unstructured domains or loops are widespread. Such a defined binding site could also provide a basis for allosteric modulation of transporter function in a diverse array of settings.

## Methods

### Cloning, expression, and purification

The *S. cerevisiae YCF1* (Yeast Cadmium Factor 1) gene was codon-optimized and cloned into the p423_GAL1 yeast expression vector as an N-terminal Flag (DYKDDDDK) and C-terminal decahistidine (10X His) tagged fusion protein (GenScript) (Supplementary Fig. 1a). The E1435Q, R-domain phosphorylation sites and interacting residues mutants were generated by site-directed mutagenesis using primers from Millipore sigma (Supplementary Table 3) and verified by sequencing (Elim Biopharmaceuticals, Inc).

For protein expression, the *S. cerevisiae* strain DSY5^38^ (Genotype MATa leu2 trp1 ura3-52 his3 pep4 prb1) was transformed with the Ycf1 expression construct and a 50 mL primary culture grown for at least 24 h at 30 °C with shaking at 200 rpm in SC-His media (0.67% w/v yeast nitrogen base without amino acids, 2% w/v glucose, and 0.08% w/v amino acid dropout mix without histidine). A secondary 750 mL culture of SC-His media was inoculated with 2% of the primary culture (15 mL) and grown under the same growth conditions for an additional 24 h prior to induction by adding YPG media (1% w/v yeast extract, 1.5% w/v peptone, and 2% w/v galactose final concentration) from a 4X YPG media stock. The culture was grown for an additional 16 h at 30 °C prior to harvesting by centrifugation at 5000 × *g* for 30 min at 4 °C.

For protein purification, harvested cells were resuspended with ice-cold lysis buffer (50 mM Tris-Cl pH 8.0, 300 mM NaCl, and cOmplete, EDTA-free protease inhibitor cocktail tablets (Roche)) at a ratio of 3.2 mL/g of cell pellet. Resuspended cells were lysed on ice by bead beating with 0.5 mm glass beads for 8 cycles consisting of 45 seconds of beating, with 5 min between cycles. Lysates were collected by vacuum filtration through a coffee filter and membranes harvested by ultracentrifugation at 112,967 × *g* for 1.5 h prior to storage at −80 °C. Membranes were solubilized in resuspension buffer (50 mM Tris-Cl pH 7.0, 300 mM NaCl, 0.5% 2,2-didecylpropane-1,3-bis-β-D-maltopyranoside (LMNG)/0.05% cholesteryl hemisuccinate (CHS) supplemented with protease inhibitor as described above) at a ratio of 15 mL/g of membrane at 4 °C for 4 h. Solubilized membranes were clarified by centrifugation at 34,155 × *g* for 30 min at 4 °C. The clarified supernatant was filtered through a 0.4 µM filter to remove the insoluble fraction and supplemented with 30 mM Imidazole pH 7.0 immediately before loading at a flow rate of 2 mL/min onto a 5 mL Ni-NTA immobilized metal affinity chromatography (IMAC) column (Bio-Rad) equilibrated in Buffer A (50 mM Tris-Cl, 300 mM NaCl, 0.01% LMNG/0.001% CHS, pH 7.0). Following loading, the column was washed with 10 column volumes (CV) of Buffer A to remove nonspecifically bound proteins then followed by a gradient of Buffer B (50 mM Tris-Cl, 300 mM NaCl, 500 mM Imidazole 0.01% LMNG/0.001% CHS, pH 7.0) consisting of the following step sizes: 6% (10CV), 10% (2CV), 16% (2CV), and 24% (2CV). Protein was eluted with 4CV of 60% buffer B and immediately diluted 10-fold with Buffer A prior to concentration and 3 rounds of buffer exchange to remove excess imidazole by centrifugation at 3095xg at 4 °C in 100 kDa cutoff concentrators (Amicon). Concentrated, buffer exchanged sample was lastly purified by size exclusion chromatography (SEC) at 4 °C by injecting sample onto a Superose 6 Increase 10/300 GL column (GE Healthcare) equilibrated in SEC buffer (50 mM Tris, 300 mM NaCl, pH 7.0) supplemented with either 0.01% LMNG/0.001%CHS or 0.06% digitonin and immediately used for biochemical assay or cryo-EM grid preparation following quantification by BCA Assay (Pierce).

### Cryo-EM grid preparation and data acquisition

Cryo-EM grids for WT and E1435Q Ycf1 were similarly prepared. Immediately following SEC purification, 5 µL of concentrated WT (15.2 mg/mL) or E1435Q (5.94 mg/mL) Ycf1 sample was applied to a CFlat-1.2/1.3-4C-T (WT Ycf1) or QF-1.2/1.3-4Au grid (E1435Q Ycf1) purchased from Electron Microscopy Sciences. Grids were placed inside of a Leica EM GP2 equilibrated to 10 °C and 80% humidity. Following a 10 s incubation, the side of the grid to which sample was applied was blotted on Whatman 1 paper (8 s for WT; 3.5 s for E1435Q), then immediately plunge frozen in liquid ethane equilibrated to −185 °C. A total of 3,262 movies were captured for WT Ycf1 using Serial EM software on a Titan Krios at 300 kV equipped with a K2 Summit detector (Gatan) at Arizona State University. For E1435Q, 8,499 movies were captured on a Titan Krios at 300 kV equipped with a K3 Summit detector (Gatan) at the Pacific Northwest Center for Cryo-EM. Movies of both WT and E1435Q Ycf1 samples were collected at 22,500X magnification with automated super resolution mode and defocus ranges of −0.5 to 2.8 µm (WT Ycf1) and −0.9 to −2.1 µm (E1435Q Ycf1). Movie frames for WT Ycf1 contained 40 frames with a per frame exposure of 1.4 electrons /Å^2^ (~56 electrons /Å^2^ total dose). Movies of E1435Q Ycf1 contained 60 frames with a per frame exposure of 0.9 electrons /Å^2^ dose rate (~54 electrons /Å^2^ total dose).

### Cryo-EM data processing

The Ycf1 E1435Q dataset was processed in RELION (3.0^39^ and 3.1^40^) and cisTEM^41^. Drift correction was performed using MotionCor2^42^ to generate an image stack with a pixel size of 1.031 Å/pixel. The contrast transfer function (CTF) was estimated for dose-weighted micrographs using CTFFIND4.1 prior to particle picking using a reconstruction of WT Ycf1 (Supplementary Fig. 2)^43^. Manual particle picking was performed on a subset of micrographs belonging to the WT Ycf1 dataset and subject to reference-free 2D Classification to generate references for automated particle picking. Following ab-initio 3D map generation and several rounds of 3D classification and 3D refinement in RELION, a ~6.0 Å resolution map of WT Ycf1 were obtained (Supplementary Fig. 2), low-pass filtered to 20 Å resolution, and used as a reference for automatic particle picking in RELION in the E1435Q mutant dataset using a 15° degree angular search (Supplementary Fig. 3). A total 2,159,582 particles were automatically picked, extracted with 4X binning resulting in a box size of 440 pixels with 4.124 Å/pixel. Multiple rounds of 2D classification were performed to remove bad particles resulting in 1,626,297 particles subject to 3D analysis in RELION following extraction with 2X binning and a box size of 300 pixels with 2.062 Å/pixel. The two major classes corresponding to two distinct states from the second round of 3D Classification were extracted with a 300 pixel box size at the full pixel size of 1.031 Å/pixel and subjected to iterative rounds of CTF refinement, Bayesian polishing, and postprocessing in RELION. To reduce alignment bias due to the presence of the detergent micelle, SIDESPLITTER refinement^44^ was implemented in later stages of 3D Refinement in RELION. A final round of alignment-free 3D Classification to remove structural heterogeneity revealed the IFnarrow and IFwide states, as well as a third state (IFtransition) in which NBD2 was poorly resolved. Maps from RELION were further refined in cisTEM and used for manual model building. ResMap was used for local resolution estimation performed on cisTEM maps^45^. A summary of the data processing workflow and final EM map quality is reported in Supplementary Figs. 2–5.

### Model building and refinement

An initial model of Ycf1 was built using the SWISS-MODEL server^46^ and PDB ID: 6JB1 as a template^47^. Manual model building was performed in COOT^48^. Iterative cycles of real-space refinement and analysis were performed in Phenix^49^ and CCP-EM modules^50,51^ were used throughout the structure-building process for map sharpening/blurring and for structure analysis. Secondary structure restraints were used extensively, and model building was guided by evolutionary couplings analysis (Supplementary Fig. 7f–g). Modrefiner was used in the early stages of refinement to help assign secondary structure and correct geometry^52^. Isolde was used to optimize model to map fit and to improve geometry^53^. Molprobity was used extensively through Phenix and through a dedicated web service to optimize geometry^54^. To maintain proper geometry, starting model restraints and harmonic restraints were used extensively in Phenix. Analysis of the Ycf1 substrate-binding cavity volume was performed using the 3 V server^55^. In this calculation, the Ycf1 NBDs were excluded in order to obtain information restricted to cavity volumes in the TMDs. A probe size of 2.5 Å was used as was previously used in assessment of a bacterial glutathione transporter^56^. Figures were prepared using UCSF ChimeraX^57^, UCSF Chimera^58^, and PyMOL^59^.

### Evolutionary coupling analysis of Ycf1

Evolutionary couplings analysis was performed using the Evcouplings package downloaded from https://github.com/debbiemarkslab/EVcouplings. Sequences were chosen automatically from the JackHMMR^60^ protocol supplied with the Evcouplings suite using the UniRef90 database. A total of 13,723 sequences were identified and coupling scores calculated with PLMC^28^. HH-suite was used to cluster similar sequences at an 80% cutoff that would otherwise skew the distribution of homologs^61^. Scores with a cutoff probability of ≥0.99% were used to identify positive interactions.

### ATPase activity assays

For evaluating ATPase activity, WT and E1435Q Ycf1 were expressed and purified as described above in buffer containing 0.01% LMNG and 0.001% CHS. ATPase rates were determined at 30 °C using an enzyme-coupled assay^62^. Each reaction consisted of 75 µL volumes containing 6.45 µg of protein in a reaction mix of 20 mM Tris-HCl pH 7.0, 10 mM MgCl_2_, 1 mM PEP, 55.7/78.03 U/mL PK/LDH, 0.3 mg/mL NADH and ATP at varying concentrations. Varying concentrations of a stock of ATP in 20 mM Tris pH 8.0 were added and final volumes were adjusted to 75 μL. Following the addition of ATP, the initial rate of NADH consumption was monitored by measuring the absorbance every minute at 340 nM for 30–45 min on a Synergy Neo2 Multi-mode Microplate Reader (BioTek). Nonlinear regression analysis of data fit with the Michaelis-Menten equation in GraphPad Prism 9 was used to generate kinetic parameters for at least three technical replicates.

Substrate-stimulated ATPase activity was measured as described above and in the presence of varying concentrations of oxidized glutathione (GSSG) (Fig. 1f and Supplementary Fig. 1e). The concentration of ATP in these experiments was held constant at 1 mM. Data for the basal ATPase activity in the absence of GSSG was subtracted from the substrate-stimulated data prior to fitting, as was performed in the study of ABCG2^63^. Data were fit using nonlinear regression in GraphPad Prism 9 to derive EC_50_ and Vmax values.

### Characterization of Ycf1 phosphorylation

Ycf1 phosphorylation was assayed by in-gel phosphoprotein staining. Dephosphorylated Ycf1 for these experiments was generated by treating SEC-purified Ycf1 (10 µg) with Lambda phosphatase [Lambda PP (2 µL), NEB] for 1 h at 30 °C in 25 µL reaction volume. Following phosphatase treatment, dephosphorylated Ycf1 was subject to a second round of SEC purification to remove excess enzyme immediately prior to use for subsequent biochemical analysis. For in-gel phosphoprotein staining, equal quantities (5 µg) of untreated Ycf1 and lambda PP (NEB) treated samples were separated on two separate 10% SDS- PAGE gels, one of which was then stained with Pro-Q Diamond Phosphoprotein Gel Stain (Thermo Scientific) following the manufacture’s protocol to detect protein phosphorylation. The second gel was simultaneously stained with Coomassie Brilliant Blue R-250 as a control for monitoring total protein levels. Full size gels are provided in Source data file.

### LC–MS/MS analysis of phosphorylated Ycf1

To measure quantitative phosphorylation of purified Ycf1, 10 µg of Ycf1 were Trypsin/LysC (Promega, Madison WI) digested with an S-Trap column (ProtiFi, Farmingdale NY) using the manufacturer’s suggested protocol following reduction with DTT and alkylation with IAA with ProteaseMax (0.1% Promega) added to the digestions. Altogether, 2500 ng of protein were loaded for each run. The LC–MS/MS analysis was performed using a Q-Exactive Plus (Thermo Fisher Scientific, San Jose, CA) mass spectrometry with an EASY-Spray nanoESI. Peptides were separated using an Acclaim Pepmap 100 trap column (75 micron ID × 2 cm from Themo Scientific) and eluted onto an Acclaim PepMap RSKC analytical column (75 micron ID x 2 cm, Thermo Scientific) with a gradient of solvent A (water and 0.1% formic acid) and solvent B (acetonitrile and 0.1% formic acid). The gradients were applied starting with 3–35% Solvent B over 90 min, then 25–50% solvent B over 20 min, 50–95% solvent B over 5 min, and a 100% solvent B for 10 min, and then 3% solvent B for 10 min.

Data were collected with a flow rate of 300 nL/min applied with a Dionex Ultimate 3000 RSLCnano system (Thermo Scientific) with data dependent scanning using Xcalibur v 4.0.27.19^64^. A survey scan at 70,000 resolution scanning mass/charge (*m*/*z*) of 350–1600 was performed with an automatic gain control target of 1e^6^ with a maximum injection time (IT) of 65 msec, then a high-energy collisional dissociation (HCD) tandem mass spectrometry (MS/MS) at 37 NCE (normalized collision energy) of the 11 highest intensity ions at 17,5000 resolution, 1.5m/z isolation width, 5e^4^ AGC, and 65msec maximum IT. Dynamic exclusion was used to select an m/z exclusion list for 30 s after single MS/MS and ions with a charge state of +1, 7, >7, unassigned, and isotopes were excluded.

Search of MS and MS/MS data were performed against the Uniprot *S. cerevisiae* protein database (https://www.uniprot.org/proteomes/UP000002311), and a database of common contaminant proteins (including trypsin, keratin – found at ftp://ftp.thegpm.org/fasta/cRAP) with Thermo Proteome Discoverer v 2.4.0.305 (Thermo Fisher Scientific). Fully tryptic peptides with up to two missed cleavage sites were considered in MS/MS spectral matches. The variable modifications considered included methionine oxidation (15.995 Da), cysteine carbamidomethylation (57.021 Da), and phosphorylation (79.966 Da) on serine, tyrosine, and threonine. XCorr score cutoffs at 95% confidence were used to identify proteins using a reverse database search^65^. Identification results from proteins and peptides were further analyzed with Scaffold Q + S v 4.11.1 (Proteome Software In., Portland OR), which integrates various search results (from Sequest, X!Tandem, MASCOT) and using Bayesian statistics to identify spectra^66^. We considered protein identification that satisfied the criteria of a minimum of two peptides with 95% confidence levels for protein and peptide.

### Limited proteolysis by trypsin protease

Fixed amounts of Lambda PP treated and untreated Ycf1 protein (6 µg) sample were incubated for 1 h on ice with vary amounts of 100 mg/mL trypsin from bovine pancreas (Sigma) at varying final concentrations (0, 2, 4,8 10, and 20 µg/mL). Samples were brought to 20 mL total volume with a buffer containing 20 mM Tris, 300 mM NaCl, and 0.05%LMNG. The reaction was stopped by adding 1 µg/mL of soybean trypsin inhibitor (Sigma) and incubating for an additional 15 min on ice. Next, 5 µL of each reaction were mixed with 1X SDS loading dye and 100 mM DTT from each reaction and separated on a 10% SDS- PAGE gel before visualizing with Coomassie Brilliant Blue R-250 staining.

### Reporting summary

Further information on research design is available in the Nature Research Reporting Summary linked to this article.

## Supplementary information

Supplementary Information

Description of Additional Supplementary Files

Supplementary Movie 1

Reporting Summary

## Data Availability

The data that support this study are available from the corresponding author upon reasonable request. The structure data generated in this study have been deposited in the Protein Data Bank database (PDB) under accession codes 7M68 and 7M69) and corresponding EM data from the EMDB with accession codes EMD-23690 and EMD-23691, respectively. Uniprot for *S. cerevisiae* proteins (https://www.uniprot.org/proteomes/UP000002311) and UniRef90 database (https://www.uniprot.org/help/uniref) for EV coupling can be accessed using provided links. The proteomics data generated in this study are provided in the Supplementary Information and Source Data file. The mass spectrometry proteomics data have been deposited to the ProteomeXchange Consortium via the PRIDE partner repository with the dataset identifier PXD031330. Source data are provided with this paper.

## Source data

Source Data
