# Supplementary material for: The structural basis for regulation of the glutathione transporter Ycf1 by regulatory domain phosphorylation
Source: Nat Commun. 2022 Mar 11;13:1278. doi: 10.1038/s41467-022-28811-w (PMC8917219; doi:10.1038/s41467-022-28811-w)
Supplement: Supplementary file 1 — Supplementary Information [file 41467_2022_28811_MOESM1_ESM.pdf]

Supplementary Information

The structural basis for regulation of the glutathione transporter Ycf1 by regulatory domain phosphorylation

Nitesh Kumar Khandelwal[1], Cinthia R. Millan[1], Samantha I. Zangari[1], Samantha Avila[2#], Dewight Williams[3], Tarjani M. Thaker[1], Thomas M. Tomasiak[1]*

Affiliations:

[1]Department of Chemistry and Biochemistry, University of Arizona; Tucson, AZ 85721

[2]Department of Biochemistry and Biophysics, University of California – San Francisco, San Francisco CA, 94158

#Present address – Duke University School of Medicine, Durham, NC, 27710

[3]Eyring Materials Center, Arizona State University; Tempe, AZ 85287

*Corresponding author. Email: tomasiak@arizona.edu

Supplementary Figures

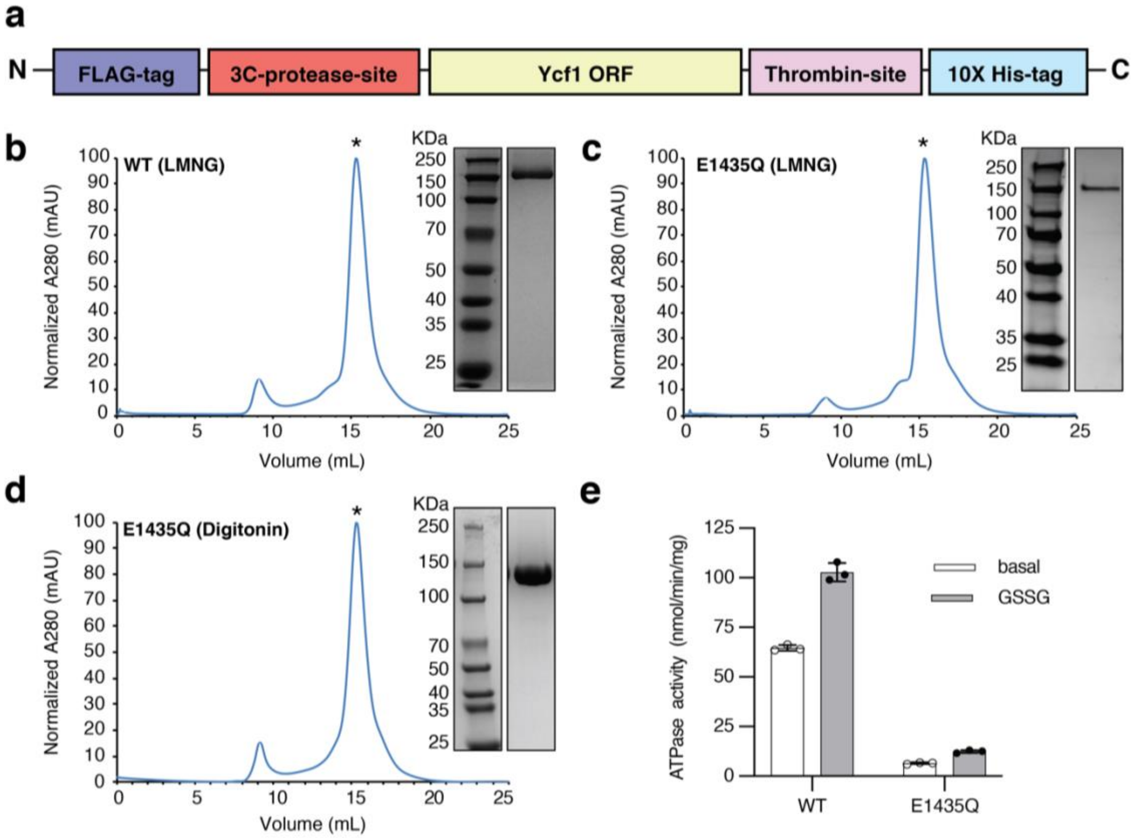

Supplementary Fig. 1. Ycf1 purification and biochemical characterization. a. Construct design for *S. cerevisiae* Ycf1 expression and purification. Representative size exclusion chromatograms (SEC) and corresponding SDS-PAGE results for purified (**b**) wild-type (WT) and (**c**) E1435Q Ycf1 in LMNG-containing buffer used for biochemical assays. Data shown are representative of multiple purifications (>3 replicates). **d.** SEC profile and corresponding SDS-PAGE result from a purification of E1435Q Ycf1 in digitonin-containing buffer used in the preparation of cryo-EM grids (single replicate). **e.** Relative ATPase activities in WT and E1435Q Ycf1 in the presence (+GSSG) and absence (basal) of 16 µM oxidized glutathione (GSSG) and 1 mM ATP. Data shown are the mean ± S.D. for n=3 (technical triplicates) and are related to main text **Fig. 1f**. Source data are provided as a Source Data file.

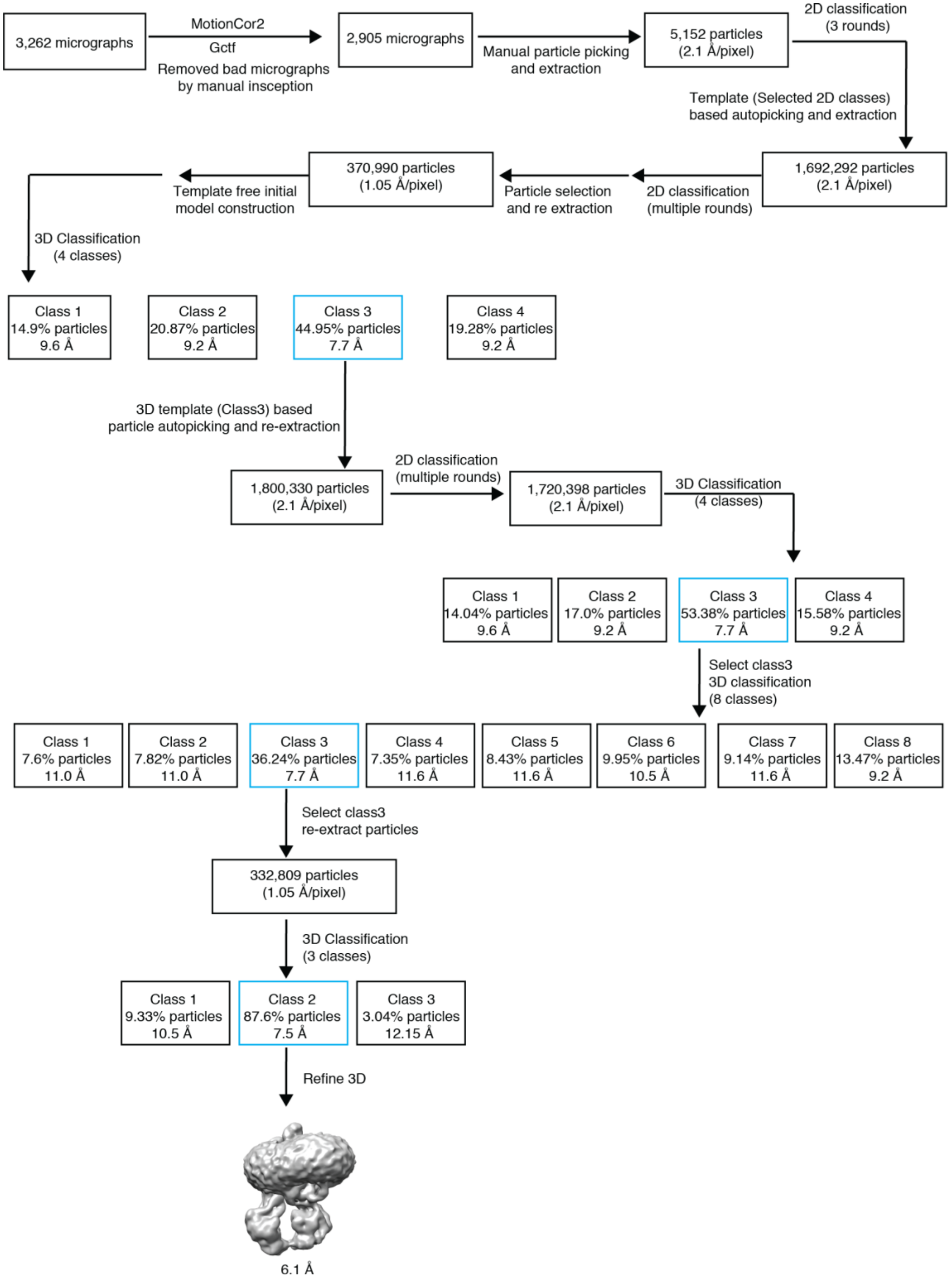

Supplementary Fig. 2. Cryo-EM data processing workflow for wild-type Ycf1. The image processing pipeline for a dataset of wild-type Ycf1 performed in RELION 3.0[1]. The resulting map was used as a template for particle picking in the E1435Q Ycf1 dataset.

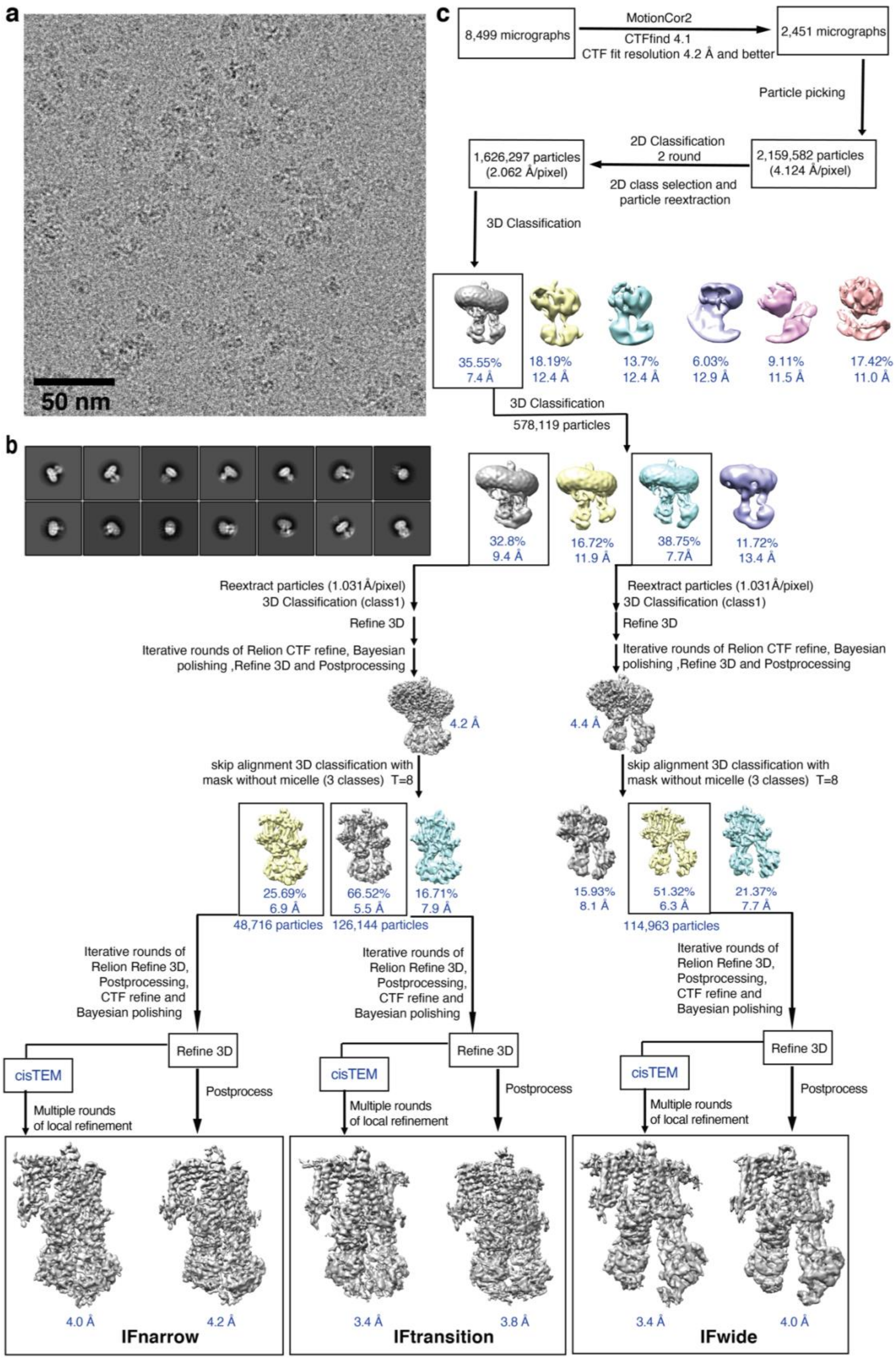
a
50 nm
b
c
8,499 micrographs
MotionCor2
CTFfind 4.1
CTF fit resolution 4.2 Å and better
2,451 micrographs
Particle picking
2,159,582 particles (4.124 Å/pixel)
2D Classification 2 round
2D class selection and particle reextraction
1,626,297 particles (2.062 Å/pixel)
3D Classification
35.55% 7.4 Å
18.19% 12.4 Å
13.7% 12.4 Å
6.03% 12.9 Å
9.11% 11.5 Å
17.42% 11.0 Å
3D Classification
578,119 particles
32.8% 9.4 Å
16.72% 11.9 Å
38.75% 7.7Å
11.72% 13.4 Å
Reextract particles (1.031Å/pixel)
3D Classification (class1)
Refine 3D
Iterative rounds of Relion CTF refine, Bayesian polishing ,Refine 3D and Postprocessing
4.2 Å
4.4 Å
skip alignment 3D classification with mask without micelle (3 classes) T=8
25.69% 6.9 Å
66.52% 5.5 Å
16.71% 7.9 Å
48,716 particles
126,144 particles
15.93% 8.1 Å
51.32% 6.3 Å
21.37% 7.7 Å
114,963 particles
Iterative rounds of Relion Refine 3D, Postprocessing, CTF refine and Bayesian polishing
Refine 3D
cisTEM
Postprocess
Multiple rounds of local refinement
4.0 Å
4.2 Å
IFnarrow
3.4 Å
3.8 Å
IFtransition
3.4 Å
4.0 Å
IFwide

Supplementary Fig. 3. Cryo-EM data processing workflow for E1435Q Ycf1. a. Representative image from a dataset consisting of 2,451 motion corrected micrographs. **b.** Gallery of representative 2D classes for particles used in 3D classification. **c.** Flowchart of map generation and refinement. Initial data were processed in RELION3.1[2]. Initials maps were refined iteratively in RELION3.1 Refine3D. In later rounds refinement was performed using the SIDESPLITTER extension for map reconstruction in RELION3.1 with the “external reconstruct” command[3]. Final rounds of local refinement were performed in cisTEM[4] using particle stacks exported from RELION3.1.

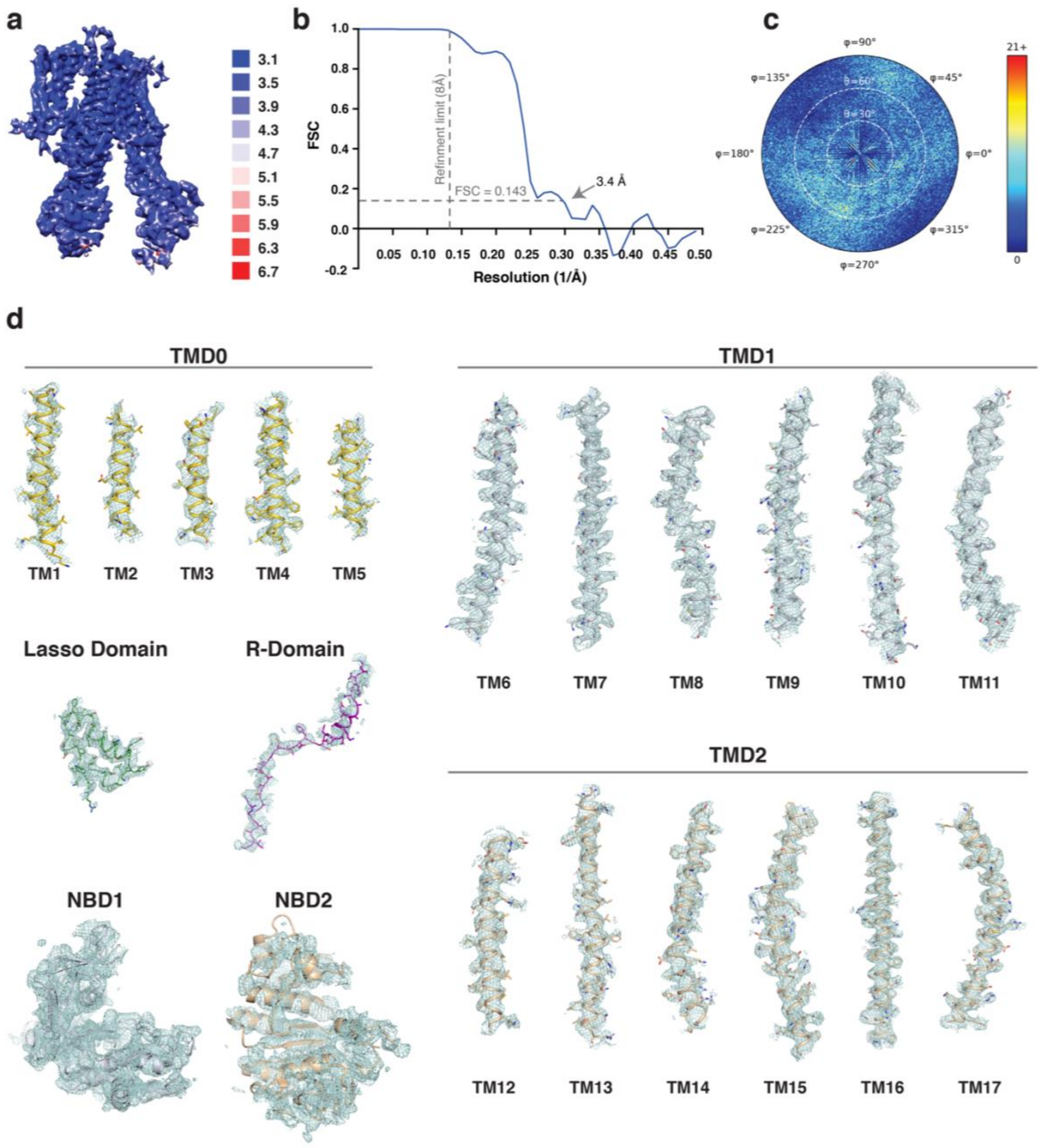

Supplementary Fig. 4. Cryo-EM map quality of the Ycf1 IFwide state. a. Refined map of the E1435Q Ycf1 IFwide conformation colored by local resolution estimated using ResMap[5]. **b.** Fourier Shell Correlation (FSC) plot from cisTEM refinement showing a global resolution of 3.4Å at a threshold of 0.143. **c.** Angular distribution of particle orientation in the final map reconstruction obtained from cisTEM. **d.** Model and corresponding densities for Ycf1 IFwide domains and transmembrane helices.

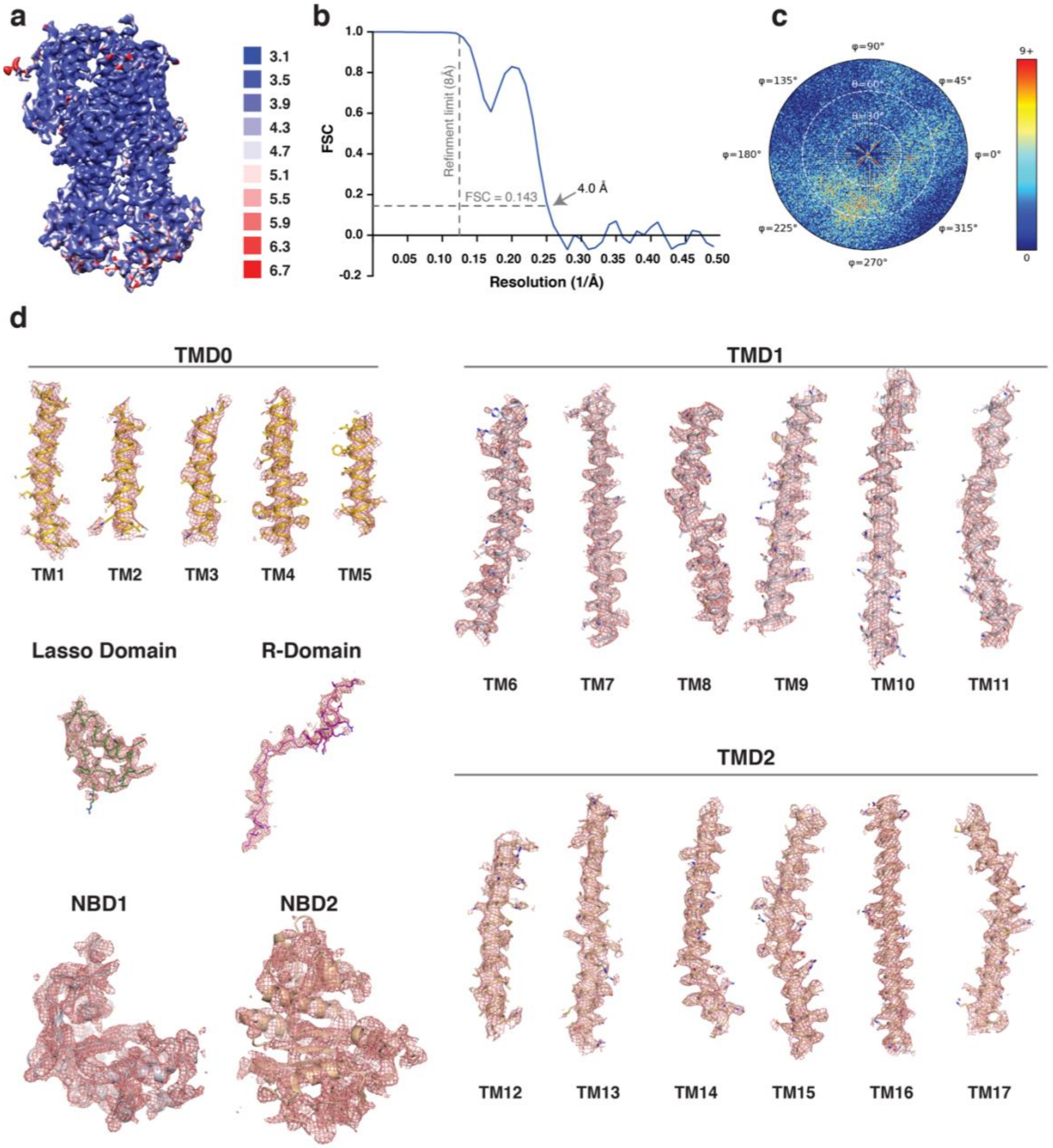

Supplementary Fig 5. Cryo-EM map quality of the Ycf1 IFnarrow state. a. Final map of E1435Q Ycf1 in the IFnarrow conformation colored by local resolution calculated using ResMap[5]. **b.** Fourier Shell Correlation (FSC) plot from cisTEM refinement showing a global resolution of 4.0Å at a threshold of 0.143, with corresponding angular distribution of particle orientations in the final map reconstruction obtained from cisTEM shown in (**c**). **d.** Model and corresponding densities for Ycf1 IFnarrow domains and transmembrane helices.

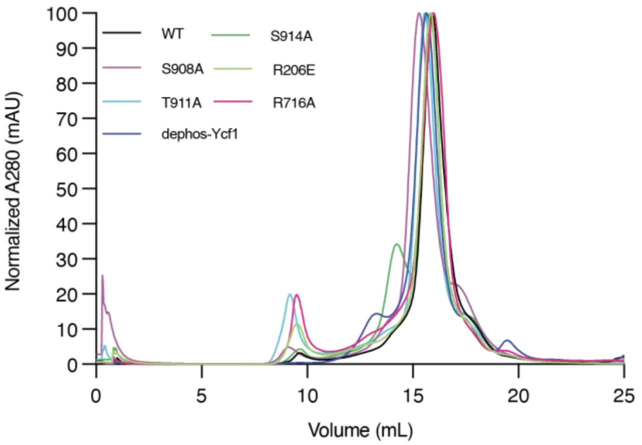

Supplementary Fig. 6. Purification of dephosphorylated and R-domain network mutants of Ycf1. Overlaid SEC profiles of WT, phospho-site mutants (S908A, T911A, S914A), R206E, R716A, and lambda phosphatase treated (dephos-Ycf1) Ycf1 samples purified in LMNG-containing buffer.

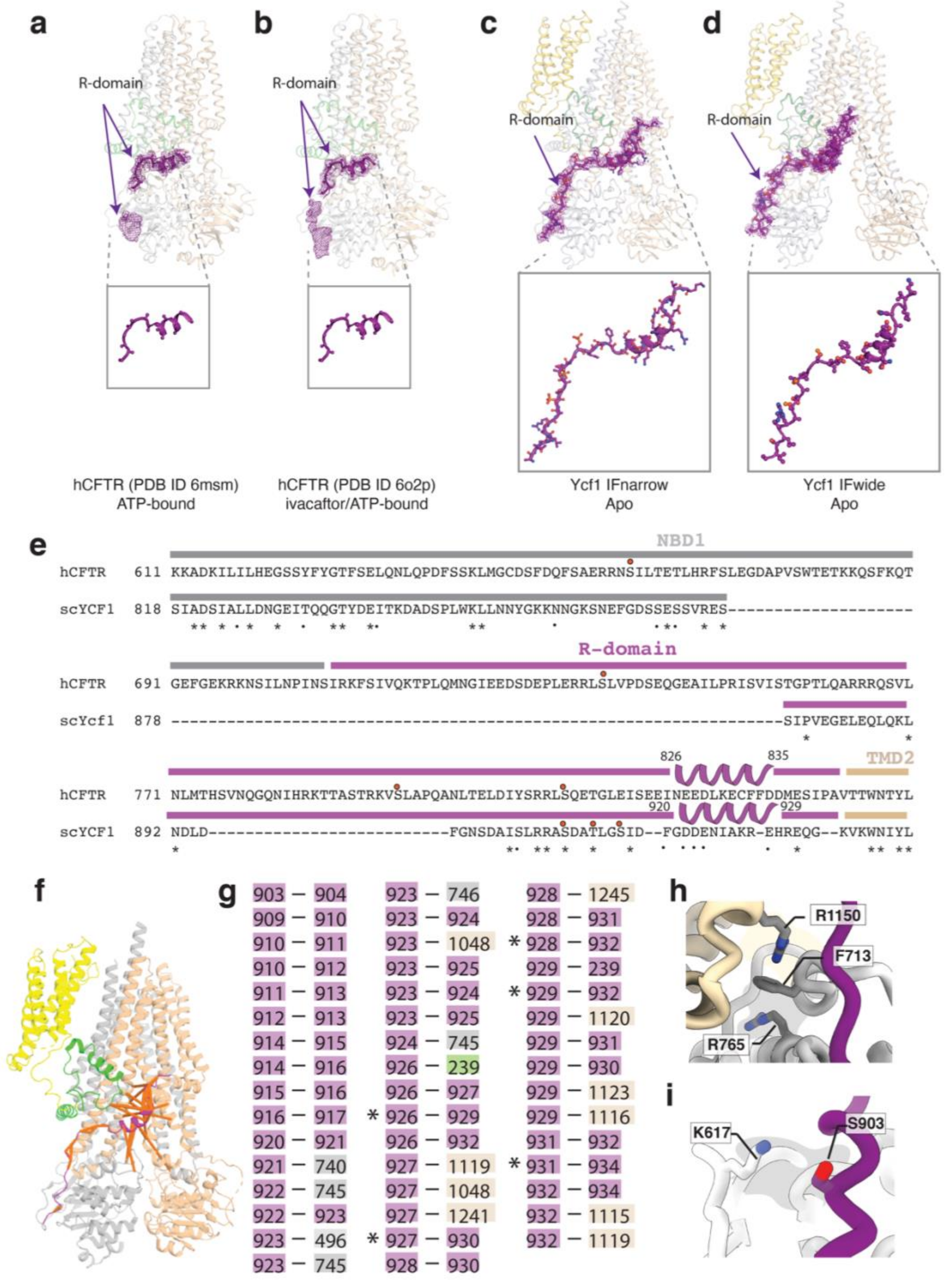

Supplementary Fig. 7. Structural comparison of the R-domain in *S. cerevisiae* Ycf1 and human CFTR. Structures of human CFTR (hCFTR) in the **a.** phosphorylated ATP-bound (PDB ID: 6MSM[6]) and **b.** ivacaftor- and ATP-bound (PDB ID: 6O2P[7]) states showing, in each case, the architecture of the helical portion of the R-domain (cartoon in purple) modeled with polyalanine residues and its corresponding density. The purple mesh represents both assigned and unassigned cryo-EM density, the latter of which the authors also attribute to the R-domain but did

not model. **c.** Architecture of the phosphorylated R-domain (purple) in the Ycf1 IFnarrow state and corresponding electron density map into which the model was built (mesh in purple). **d.** The same representation as in (**c**) for the Ycf1 IFwide state. Highlighted below panels (**a-d**) are the amino acid assignments of the R-domain secondary structure in each model. The lasso domain is shown in green, TMD0 in yellow, TMD1 in light grey, and TMD2 in wheat. **e.** Sequence comparison of the hCFTR and Ycf1 R-domains from an alignment performed using AlignMe[8] and manually adjusted. Residues phosphorylated in both structures are denoted with an orange circle above the corresponding site. Sequence similarity is higher in the C-terminus of the R-domain as compared to the N-terminus. **f.** Network of evolutionary couplings between residues comprising the R-domain (901-935) are shown as orange bands in the structure of Ycf1 IFnarrow colored the same as in (**a-d**). **g.** Annotated list of evolutionary couplings shown in (**f**) between residue pairs colored by the domain in which they reside (same as in **a-d**), with important helical interactions denoted with an asterisk. **h.** Closeup of the interaction network between F713, R1150, R765 in IFnarrow. **i.** Residue K617 in regulatory insertion (residues 615-643) in proximity to S903 in the R-domain, another residue that is known to be phosphorylated in Ycf1.

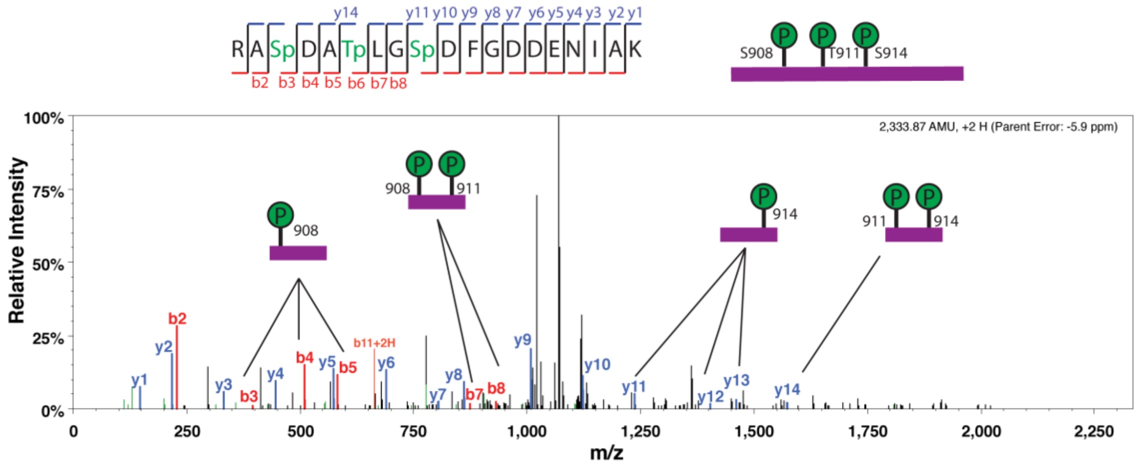

Supplementary Fig. 8. Representative mass spectrum of Ycf1 showing phosphorylation of residues S908, T911, and S914. The identified b and y ions from the entire peptide are denoted on top of the peptide sequence with y ions in blue and b ions in red. Phosphorylated residues are colored green in the sequence. Diagrams of the phosphorylated peptides with green spheres representing phosphorylation sites are shown. Black lines from each fragment denote the b and y ions specific to them. Source data are provided as a Source Data file.

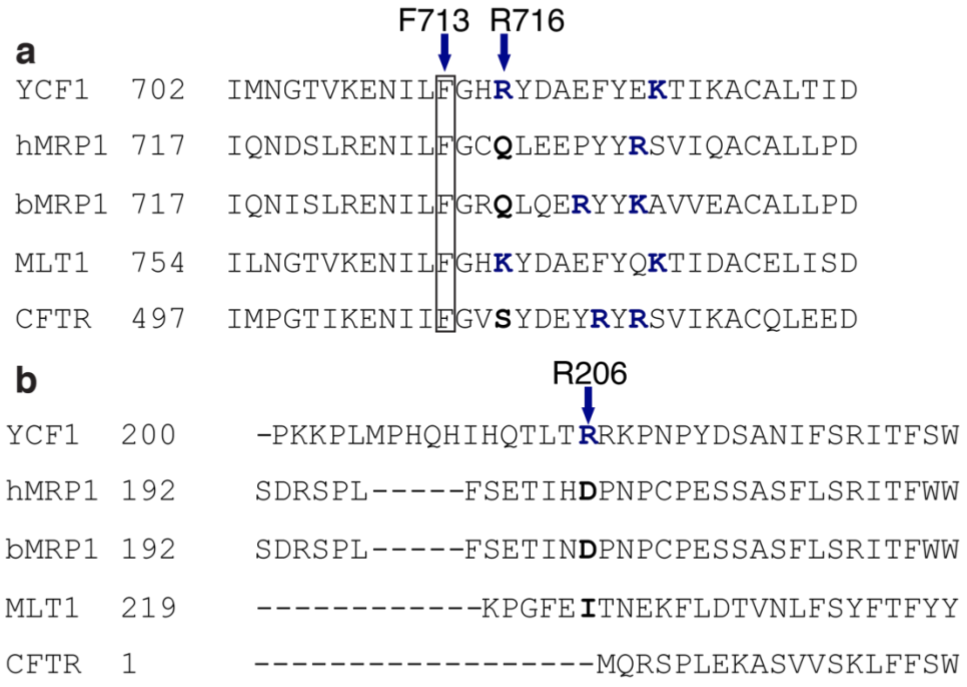

Supplementary Fig. 9. Sequence alignment of ABCC family members showing conservation of residues interacting with R-domain phosphorylated residues (S908, T911 and S914). a. R716 in Ycf1 interacts with phosphorylated conserved residues (S908 and T911) (Fig2.B). Other members at this position are either polar or positively charged residues and several positively charged residues are located nearby. Residues at the 716 position are shown in bold and positively charged residues are shown in blue. The position of F713 (F508 in CFTR) are shown nearby and are highlighted with a black box. **b.** The conservation of R206 is shown. No conservation or pattern is observed.

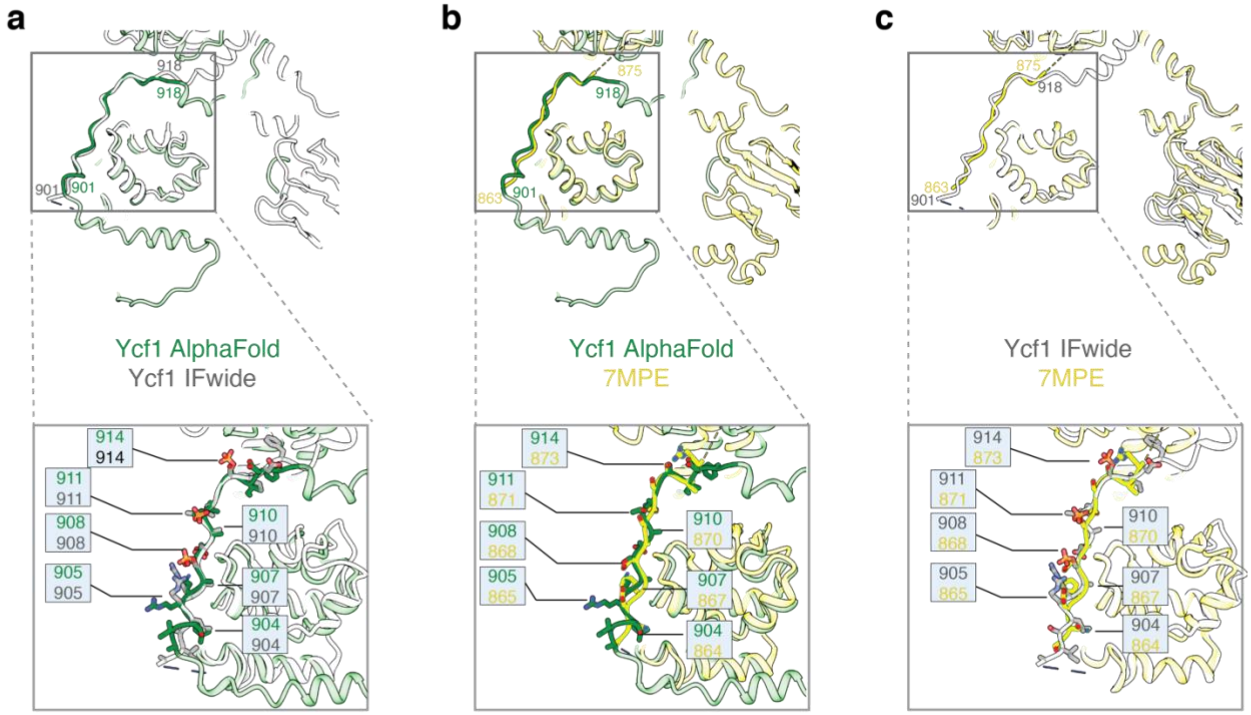

Supplementary Fig. 10. Comparison of R-domain architecture in multiple Ycf1 models. Overlays of the **a.** Ycf1 AlphaFold2 model (green) with Ycf1 IFwide (gray), **b.** Ycf1 AlphaFold2 model (green) and PDB ID 7MPE[9] (yellow), and **c.** Ycf1 IFwide (gray) and 7MPE (yellow). Details of the amino acid composition of a segment of the R-domain in equivalent positions (IFwide: 901-914, AlphaFold2: 901-914, 7MPE: 864-873) are shown in the panels below, with side chains shown as sticks (colored the same as in the cartoon representation in panel above) to highlight similarities or differences between each.

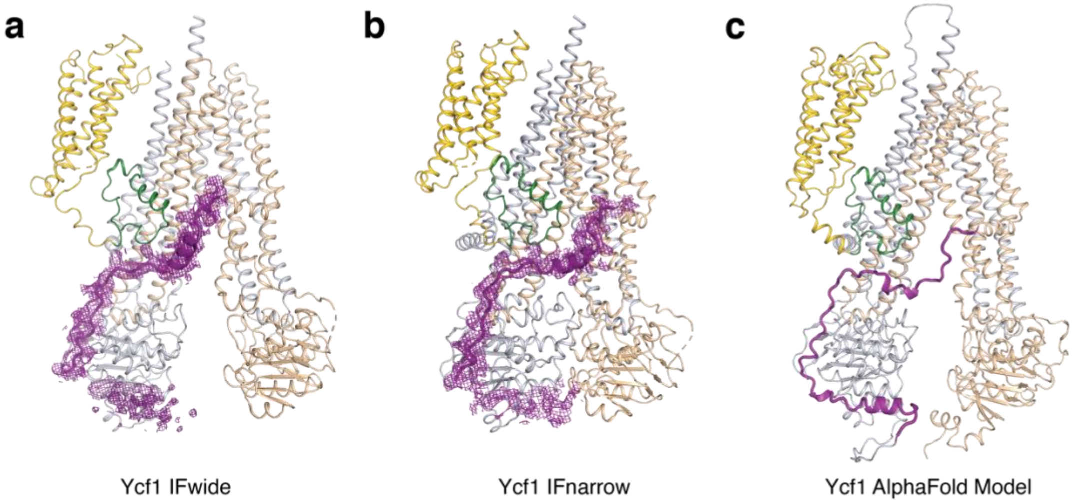

Supplementary Fig. 11. Comparison of Cryo-EM structures of IFwide and IFnarrow Ycf1 to the AlphaFold2 prediction. Conserved regions in the **a.** IFwide and **b.** IFnarrow Ycf1 cryo-EM structures are shown colored the same as in **c.** the Ycf1 AlphaFold2 prediction in the same orientation as IFwide and IFnarrow to highlight the conservation of the R-domain architecture (purple) in all three. The cryo-EM density corresponding to the R-domain in IFwide and IFnarrow is shown as a purple mesh, and closely matches the topology of the R-domain in (**c**).

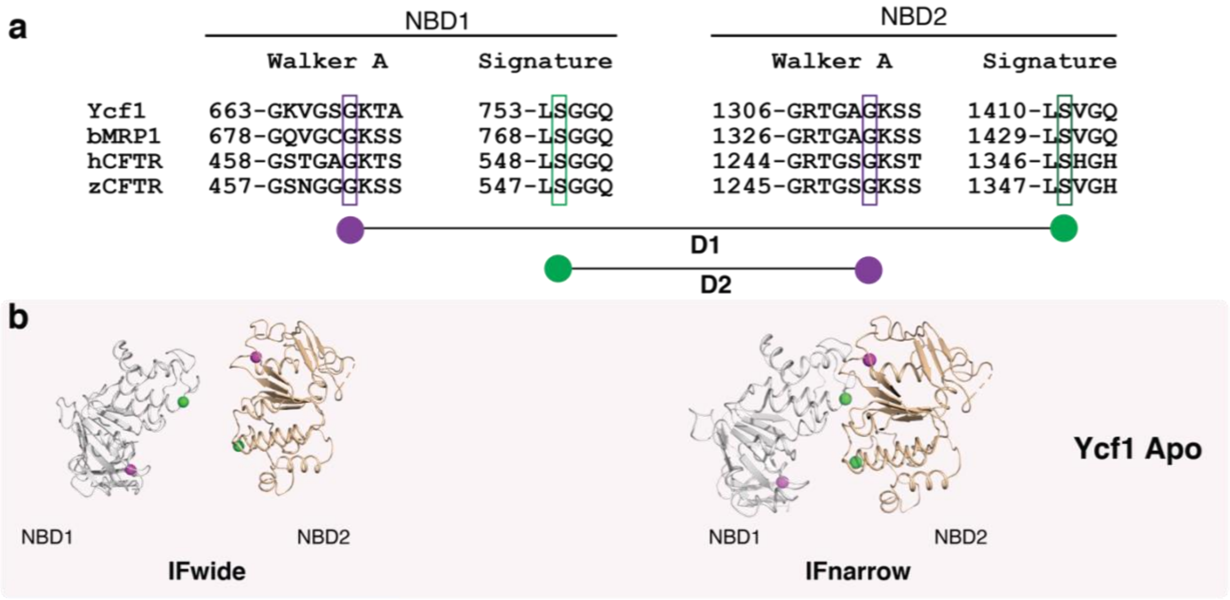

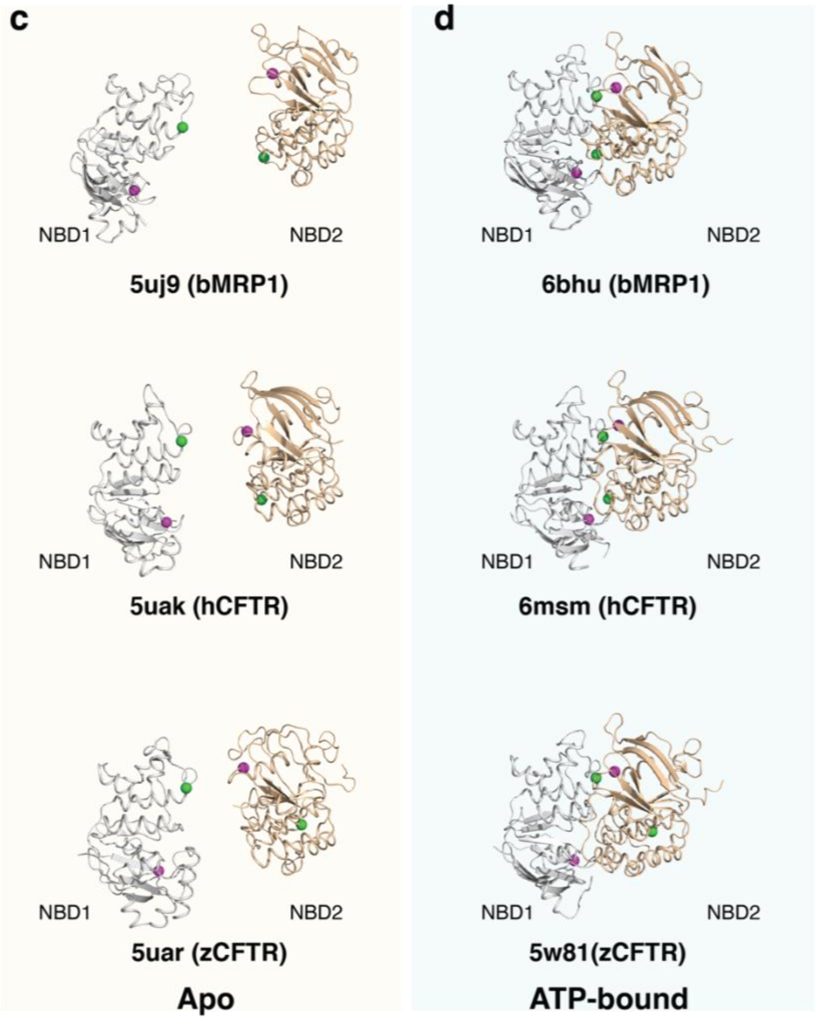

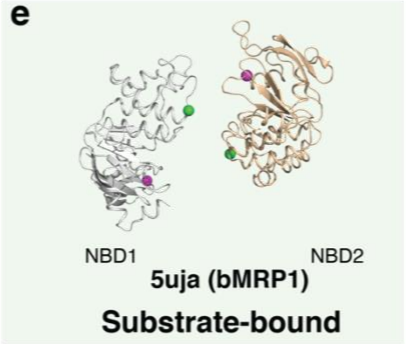

f

Structure	D1 (Å)	D2 (Å)
Ycf1(IFwide)	35.4	28.8
5uj9 (bMRP1)	42.9	34.8
5uak (hCFTR)	30.6	21.0
5uar (zCFTR)	47.2	19.4
Ycf1(IFnarrow)	20.9	17.8
5uja (bMRP1)	27.8	24.0
6bhu (bMRP1)	8.6	9.0
6msm (hCFTR)	9.0	8.7
5w81(zCFTR)	26.0	8.6

Supplementary Fig.12. Structural comparison of Ycf1 NBD architecture to related C family ABC transporters. a. Sequence alignment highlighting residues of the Walker A and signature motifs from NBD1 and NBD2 at the NBD dimer interface in Ycf1, bovine MRP1 (bMRP1), human CFTR (hCFTR), and zebrafish CFTR (zCFTR). The interatomic distances between the conserved glycine (purple sphere) of the Walker A motif in NBD1 and conserved serine (green sphere) of the signature motif in NBD2 are denoted as D1. The interatomic distances between the conserved serine (green sphere) of the signature motif in NBD1 and conserved glycine (purple sphere) of the Walker A motif in NBD2 is denoted as D2. **b-e.** Bottom view of NBDs in (**b**) Ycf1 IFwide and IFnarrow structures, (**c**) bMRP1 (PDB ID: 5UJ9[10], hCFTR (PDB ID: 5UAK[11]) and zCFTR (PDB ID: 5UAR[12]) in the apo conformations. (**d**) NBDs bMRP1 (PDB ID: 6BHU[13]), hCFTR (PDB ID: 6MSM[6]) and zCFTR (PDB ID: 5W81[14]) in the ATP-bound conformations. (**e**) bMRP1 (PDB ID: 5UJA[10], in the leukotriene C4 substrate-bound conformation. **f.** Distance measurements between the residues described in (**a**) in the representative ABCC family transporters shown.

Supplementary Tables

Supplementary Table 1. Cryo-EM data collection and refinement statistics

**Data collection**		
Microscope	ThermoFisher Titan Krios	
Acceleration voltage	300kV	
Detector	Gatan K3	
Image pixel size	1.031 Å	
Defocus range	-0.9 to -2.1 µm	
Electron exposure	~54 electrons /Å^2	
Number of frames	60	
Number of micrographs	8,499	
**Image Processing**		
	**IFwide**	**IFnarrow**
No. of particles in final reconstruction	114,963	48,716
Symmetry	C1	C1
Final box size (pixels)	300	300
Global resolution (RELION map)	4.0 Å	4.2 Å
Global resolution (cisTEM map)	3.4 Å	4.0 Å
FSC threshold	0.143	0.143
Refinement		
Atoms	21,797	21,822
Residues	1,390	1,390
Water	0	0
Supplied Resolution (Å)	3.42	4.0
B-factors (Å^2)		
Iso/Aniso (#)	10883/0	10887/0
Protein (min/max/mean)	85.10/214.51/146.40	86.62/214.73/147.63
Bonds (RMSD)		
Length (Å)	0.005	0.005
Angles (°)	0.855	0.736
**Validation**		
MolProbity score	1.19	1.20
Clash score	0.82	1.05
Ramachandran plot (%)		
Outliers	0	0
Allowed	5.11	5.76
Favored	94.89	94.24
Rotamer outliers (%)	1.29	0.60

**Model vs. Data**		
CC (mask)	0.70	0.70
CC (box)	0.52	0.51
CC (peaks)	0.34	0.32
CC (volume)	0.70	0.71
PDB ID	7M69	7M68

Supplementary Table 2. Ycf1 phosphorylation spectrum counts.

P39109|YCFI_YEAST) Metal resistance protein YCF1 OS=*Saccharomyces cerevisiae* (*strain ATCC 204508 / S288c*) GN=YCF1 PE=1 SV=2				
**Site**	**Modification**	**Best A score**	**Localization Probability**	**TT_3112287_YCF1_Soln_2500ng_75min**
S251	Phospho	58.42	0.99999857	8
T732	Phospho	109.04	1	1
S846	Phospho	45.06	0.99999803	1
S869	Phospho	27.96	0.99998724	1
S878	Phospho	24.95	0.99681276	1
S903	Phospho	104.99	1	26
S908	Phospho	1,000.00	1	34
T911	Phospho	1,000.00	1	19
S914	Phospho	1,000.00	1	25
S1268	Phospho	68.14	1	1
END OF FILE				

References

1. Zivanov, J. *et al.* New tools for automated high-resolution cryo-EM structure determination in RELION-3. *Elife* **7**, e42166 (2018).
2. Zivanov, J., Nakane, T. & Scheres, S. H. W. Estimation of high-order aberrations and anisotropic magnification from cryo-EM data sets in RELION-3.1. *IUCrJ* **7**, 253–267 (2020).
3. Ramlaul, K., Palmer, C. M., Nakane, T. & Aylett, C. H. S. Mitigating local over-fitting during single particle reconstruction with SIDESPLITTER. *J. Struct. Biol.* **211**, 107545 (2020).
4. Grant, T., Rohou, A. & Grigorieff, N. cisTEM, user-friendly software for single-particle image processing. *Elife* **7**, e35383 (2018).
5. Kucukelbir, A., Sigworth, F. J. & Tagare, H. D. Quantifying the local resolution of cryo-EM density maps. *Nat. Methods* **11**, 63–65 (2014).
6. Zhang, Z., Liu, F. & Chen, J. Molecular structure of the ATP-bound, phosphorylated human CFTR. *Proc. Natl. Acad. Sci. U. S. A.* **115**, 12757–12762 (2018).
7. Liu, F. *et al.* Structural identification of a hotspot on CFTR for potentiation. *Science* **364**, 1184–1188 (2019).
8. Stamm, M., Staritzbichler, R., Khafizov, K. & Forrest, L. R. AlignMe - A membrane protein sequence alignment web server. *Nucleic Acids Res.* **42**, W246–W251 (2014).
9. Bickers, S. C., Benlekbir, S., Rubinstein, J. L. & Kanelis, V. Structure of Ycf1p reveals the transmembrane domain TMD0 and the regulatory region of ABCC transporters. *Proc. Natl. Acad. Sci.* **118**, e2025853118 (2021).
10. Johnson, Z. L. & Chen, J. Structural Basis of Substrate Recognition by the Multidrug Resistance Protein MRP1. *Cell* **168**, 1075-1085.e9 (2017).
11. Liu, F. *et al.* Molecular Structure of the Human CFTR Ion Channel. *Cell* **169**, 85–95 (2017).
12. Zhang, Z. & Chen, J. Atomic Structure of the Cystic Fibrosis Transmembrane Conductance Regulator. *Cell* **167**, 1586-1597.e9 (2016).
13. Johnson, Z. L. & Chen, J. ATP Binding Enables Substrate Release from Multidrug Resistance Protein 1. *Cell* **172**, 81-89.e10 (2018).
14. Zhang, Z., Liu, F. & Chen, J. Conformational Changes of CFTR upon Phosphorylation and ATP Binding. *Cell* **170**, 483-491.e8 (2017).
