# Supplementary material for: The structural basis for regulation of the glutathione transporter Ycf1 by regulatory domain phosphorylation
Source: Nat Commun. 2022 Mar 11;13:1278. doi: 10.1038/s41467-022-28811-w (PMC8917219; doi:10.1038/s41467-022-28811-w)
Supplement: Supplementary file 2 — Description of Additional Supplementary Files [file 41467_2022_28811_MOESM2_ESM.pdf]

Description of Additional Supplementary Files

File Name: Supplementary Movie 1
Description: Movie representing conformation changes of Ycf1 from IFwide to IFnarrow stage.
