# Supplementary material for: The structural basis for regulation of the glutathione transporter Ycf1 by regulatory domain phosphorylation
Source: Nat Commun. 2022 Mar 11;13:1278. doi: 10.1038/s41467-022-28811-w (PMC8917219; doi:10.1038/s41467-022-28811-w)
Supplement: Supplementary file 4 — Reporting Summary [file 41467_2022_28811_MOESM4_ESM.pdf]

nature research

Corresponding author(s): Thomas M . Tomasiak

Last updated by author(s): 2022-01-31

Reporting Summary

Nature Research wishes to improve the reproducibility of the work that we publish. This form provides structure for consistency and transparency in reporting. For further information on Nature Research policies, see our Editorial Policies and the Editorial Policy Checklist.

Statistics

For all statistical analyses, confirm that the following items are present in the figure legend, table legend, main text, or Methods section.

n/a	Confirmed	
☐	☒	The exact sample size (*n*) for each experimental group/condition, given as a discrete number and unit of measurement
☐	☒	A statement on whether measurements were taken from distinct samples or whether the same sample was measured repeatedly
☒	☐	The statistical test(s) used AND whether they are one- or two-sided<br>*Only common tests should be described solely by name; describe more complex techniques in the Methods section.*
☒	☐	A description of all covariates tested
☒	☐	A description of any assumptions or corrections, such as tests of normality and adjustment for multiple comparisons
☐	☒	A full description of the statistical parameters including central tendency (e.g. means) or other basic estimates (e.g. regression coefficient) AND variation (e.g. standard deviation) or associated estimates of uncertainty (e.g. confidence intervals)
☒	☐	For null hypothesis testing, the test statistic (e.g. *F*, *t*, *r*) with confidence intervals, effect sizes, degrees of freedom and *P* value noted<br>*Give P values as exact values whenever suitable.*
☒	☐	For Bayesian analysis, information on the choice of priors and Markov chain Monte Carlo settings
☒	☐	For hierarchical and complex designs, identification of the appropriate level for tests and full reporting of outcomes
☒	☐	Estimates of effect sizes (e.g. Cohen's *d*, Pearson's *r*), indicating how they were calculated

Our web collection on statistics for biologists contains articles on many of the points above.

Software and code

Policy information about availability of computer code

Data collection	Serial EM was used for automated data collections.
Data analysis	RELION3.0, RELION 3.1, cisTEM 1.0, MotionCor2, CTFFIND4.1, SIDESPLITTER 1.3, SIDESPLITTER beta, ResMap-1.1.4, SWISS-MODEL server 1.0, COOT 0.8, COOT 0.9, Phenix1.17, Phenix 1.19, CCP-EM 1.0 modules, Modrefiner, Isolde 0.93, Isolde1.1 , Molprobity4.02, 3V server, UCSF ChimeraX .93 , UCSF ChimeraX1.1, UCSF Chimera 1.13, Pymol 2.4 and Pymol2.5, Graphpad prism 9, AlphaFold2, EV-coupling (https//github.com/debbiemarkslab/EVcouplings.), Xcaliburv 4.0.27.19, Scaffold Q+S v 4.11.1 ,Thermo Proteome Discover v 2.4.0.305

For manuscripts utilizing custom algorithms or software that are central to the research but not yet described in published literature, software must be made available to editors and reviewers. We strongly encourage code deposition in a community repository (e.g. GitHub). See the Nature Research guidelines for submitting code & software for further information.

Data

Policy information about availability of data

All manuscripts must include a data availability statement. This statement should provide the following information, where applicable:

- Accession codes, unique identifiers, or web links for publicly available datasets
- A list of figures that have associated raw data
- A description of any restrictions on data availability

All of the date presented here have been uploaded to the Protein Data Bank (PDB codes: 7M68 and 7M69) and the EMDB (EMD-23690 and EMD-23691). The mass spectrometry proteomics data have been deposited to the ProteomeXchange Consortium via the PRIDE partner repository with the dataset identifier PXD031330. Source data are provided with this paper.

Field-specific reporting

Please select the one below that is the best fit for your research. If you are not sure, read the appropriate sections before making your selection.

☒ Life sciences ☐ Behavioural & social sciences ☐ Ecological, evolutionary & environmental sciences

For a reference copy of the document with all sections, see nature.com/documents/nr-reporting-summary-flat.pdf

Life sciences study design

All studies must disclose on these points even when the disclosure is negative.

Sample size
Sample size calculations were not performed. The amount of protein used was based on protein used in field earlier for ATPase assay and found sufficient based on assay data obtained. All the ATPase assays were performed at least in technical triplicate and mentioned in figure legends

Data exclusions
All raw cryo-EM data were processed through the Relion3.0 and Relion3.1 pipelines. Micrographs were excluded based on poor resolution estimates using CTFFIND which often correlated with heavy ice contamination. Particle selection was performed following 2D and 3D classification in Relion and judging by predicted resolution and visual inspection of classes.

Replication
All experiments were performed at least in triplicate (technical replicates) and information provided in figure legends. All attempts were successful and gave consistent results.

Randomization
Particles were randomized into two half sets following the standard automated Relion protocol.

Blinding
Researchers were not blinded to group allocation. All internal assignment to different classes were performed internally by the reconstruction software (Relion).

Reporting for specific materials, systems and methods

We require information from authors about some types of materials, experimental systems and methods used in many studies. Here, indicate whether each material, system or method listed is relevant to your study. If you are not sure if a list item applies to your research, read the appropriate section before selecting a response.

Materials & experimental systems

n/a	Involved in the study	
☒	☐	Antibodies
☐	☒	Eukaryotic cell lines
☒	☐	Palaeontology and archaeology
☒	☐	Animals and other organisms
☒	☐	Human research participants
☒	☐	Clinical data
☒	☐	Dual use research of concern

Methods

n/a	Involved in the study	
☒	☐	ChIP-seq
☒	☐	Flow cytometry
☒	☐	MRI-based neuroimaging
